# Advanced immunophenotyping: A powerful tool for immune profiling, drug screening, and a personalized treatment approach

**DOI:** 10.3389/fimmu.2023.1096096

**Published:** 2023-03-24

**Authors:** Teresa Preglej, Marie Brinkmann, Günter Steiner, Daniel Aletaha, Lisa Göschl, Michael Bonelli

**Affiliations:** Division of Rheumatology, Department of Internal Medicine III, Medical University of Vienna, Vienna, Austria

**Keywords:** immunophenotyping, drug screening, PBMC (peripheral blood mononucleated cells), T cells, flow cytometry

## Abstract

Various autoimmune diseases are characterized by distinct cell subset distributions and activation profiles of peripheral blood mononuclear cells (PBMCs). PBMCs can therefore serve as an ideal biomarker material, which is easily accessible and allows for screening of multiple cell types. A detailed understanding of the immune landscape is critical for the diagnosis of patients with autoimmune diseases, as well as for a personalized treatment approach. In our study, we investigate the potential of multi-parameter spectral flow cytometry for the identification of patients suffering from autoimmune diseases and its power as an evaluation tool for *in vitro* drug screening approaches (advanced immunophenotyping). We designed a combination of two 22-color immunophenotyping panels for profiling cell subset distribution and cell activation. Downstream bioinformatics analyses included percentages of individual cell populations and median fluorescent intensity of defined markers which were then visualized as heatmaps and in dimensionality reduction approaches. *In vitro* testing of epigenetic immunomodulatory drugs revealed an altered activation status upon treatment, which supports the use of spectral flow cytometry as a high-throughput drug screening tool. Advanced immunophenotyping might support the exploration of novel therapeutic drugs and contribute to future personalized treatment approaches in autoimmune diseases and beyond.

## 1 Introduction

Autoimmunity is defined by an imbalance of activating and inhibitory cascades of the immune system resulting in loss of self-tolerance (1). So far, the diagnosis of autoimmune diseases relies on the presence of certain autoantibodies, such as the anti-citrullinated protein antibody (ACPA) or rheumatoid factor (RF) in rheumatoid arthritis (RA). Although, autoantibodies are used as biomarkers indicating accurate disease classification and treatment response (2, 3), their interpretability displays several shortcomings. Over the past decade, markers found in serum, plasma, and whole blood have been tested for their potential as biomarkers in autoimmunity (4–6). Previously, cellular biomarkers from Peripheral blood mononuclear cells (PBMCs) indicative of autoimmune disorders have moved into the focus of science (7–15). PBMCs consist of many different cell types with varying frequencies. They function as drivers and regulators of physiological and pathological immune responses, which can lead to autoimmunity and inflammation (16). Alterations in PBMC subsets, and especially in T helper (Th) cells are characteristic of various autoimmune disorders and contribute to the pathogenesis, for instance of RA, multiple sclerosis (MS), or systemic lupus erythematosus (SLE) (8, 11, 12). Several studies demonstrated, that patients suffering from autoimmune disease conditions exhibit elevated levels of antibody-secreting B cells and CD4^+^ memory T cells, respectively, compared to healthy individuals (17–19). Increased frequencies of distinct CD4^+^ T cell subsets, such as Th1, Th17, T follicular helper cells (Tfh), regulatory T cells (Treg), and CD4^+^CD25^-^Foxp3^+^ T cells, have been linked to aberrant immune responses (7–15). Hence, certain PBMC compartments might constitute promising cellular biomarkers for the application as clinical parameters, as shown for CD19^+^ B cell numbers as an indicator for RA relapse after B cell depletion therapy using Rituximab (20–23). Further, the expression ratio of activation and inhibitory surface receptors on immune cells can be used for the identification of patients with autoimmune diseases (24). A comprehensive understanding of the architecture and inflammatory potential of immune cells is critical for the analysis of autoimmune disorders (2, 3). In this regard, it is essential to elucidate the homeostatic composition of the immune landscape, and also the activation status. Thus, the characterization of PBMCs that are easily accessible can serve as a tool for the diagnosis of patients, the prediction of treatment responses, or the verification of potential personalized treatment approaches.

Over the past decades, flow cytometry has been applied to identify the presence and individual proportions of specific leukocyte populations termed immunophenotyping. In a clinical context, immunophenotyping is utilized for the identification of autoimmune diseases, autoimmune deficiencies, and hematological malignancies (25, 26). Therefore, flow cytometry constitutes the most successful state-of-the-art method to detect extracellular and intracellular proteins of interest on a single-cell level. Until recently, the binding of fluorophore-labeled antibodies to their targets enabled fast detection of protein levels and easy downstream analysis of the generated data. However, conventional flow cytometry suffers from the limited number of implemented detectors, resulting in a fluorescent overlap of the different fluorescent dyes and compensation inaccuracies. Therefore, the cap of available fluorophore combinations results in diminished complexity of staining panels. Recent advancements constitute the establishment of spectral flow cytometry. These novel multi-detector approaches allow for the measurement of the entire emission spectrum of individual fluorophores across all lasers. The full spectrum technology produces a distinct spectral fingerprint for every fluorophore, enabling the mathematical separation of dyes with almost similar emission peaks (27–29), which is inaccessible using a conventional flow cytometry approach. An additional advantage over the conventional system constitutes the possibility to extract the autofluorescence of cells, resulting in increased population resolution of samples. Further, spectral flow cytometry displays an elevated separation performance of populations achieved by a reduction of the signal spreading error (30, 31). Thus, the full spectrum technology allows for the establishment of flexible and large staining panels that are capable of expanding the application range by additional, highly overlapping fluorophores (30, 31). Recently, the continuously evolving field of spectral flow cytometry was able to establish a 40-color assay (32). Solely mass cytometry, a single-cell proteomics approach using metal isotope labeled antibodies, possesses the ability to acquire more markers in one staining panel. However, this technique suffers from high costs and space requirements, as well as the need for professional expertise to operate the measurements (33). In contrast, the full spectrum technology, using sophisticated panels composing more than 20 fluorochromes, can be established in the majority of laboratories. In this context, spectral flow cytometry can serve as a powerful broad screening tool, for instance assessing the entire composition of PBMCs in a single measurement (32, 34–37). One potential application beyond the use as a diagnostic tool might constitute multi-parameter drug screening. So far, drug screening assays were applied on tumor cells solely assessing the toxicity of the compounds. However, there is a desperate urge to identify more sophisticated approaches. Novel methodologies, either assess drug-induced transcriptional changes or investigate alterations in cell-cell contact by high-content microscopy (38, 39). These methods either lack cell subset-specific information or suffer from a limited number of measurable variables (38, 39).

The identification of new therapeutic agents for immune-mediated diseases requires insight into their mode-of-action on distinct immune cell compartments. Therefore, in the presented study we introduce advanced immunophenotyping, a novel approach linking an *in vitro* drug screening assay, multi-color spectral flow cytometry, and comprehensive downstream analysis using bioinformatics tools. The major advantage over previously published flow cytometry studies is the combination of a detailed identification of certain PBMC subsets and a functional characterization of specific drug-induced alterations. We established two 22-color staining panels to determine dynamics in the expression of activation and inhibitory receptors among all major PBMC populations, such as T cells, B cells, monocytes, natural killer (NK) cells, and dendritic cells (DCs), as well as the respective subpopulations. Additionally, we demonstrated the applicability of the established panels to assess changes in the composition and activation status of distinct PBMC populations in an *in vitro* drug screening assay, as well as for the identification of patients suffering from autoimmune diseases.

In summary, we present a spectral flow cytometry-based high-throughput approach for the detection of drug-induced effects in PBMCs and suggest tools for downstream bioinformatics analysis. Future studies are needed to validate the potential of this technique to determine promising therapeutic biomarkers. In addition, we demonstrate the capacity of immunophenotyping to serve as a functional tool for the identification of patients suffering from autoimmune diseases. Thus, the presented approach enables fast and broad screening not only of patients, but also of different immunomodulatory drugs providing comprehensive insights into their efficacy and mode-of-action.

## 2 Results

### 2.1 Guidance for panel design strategies

**Figure 1:**
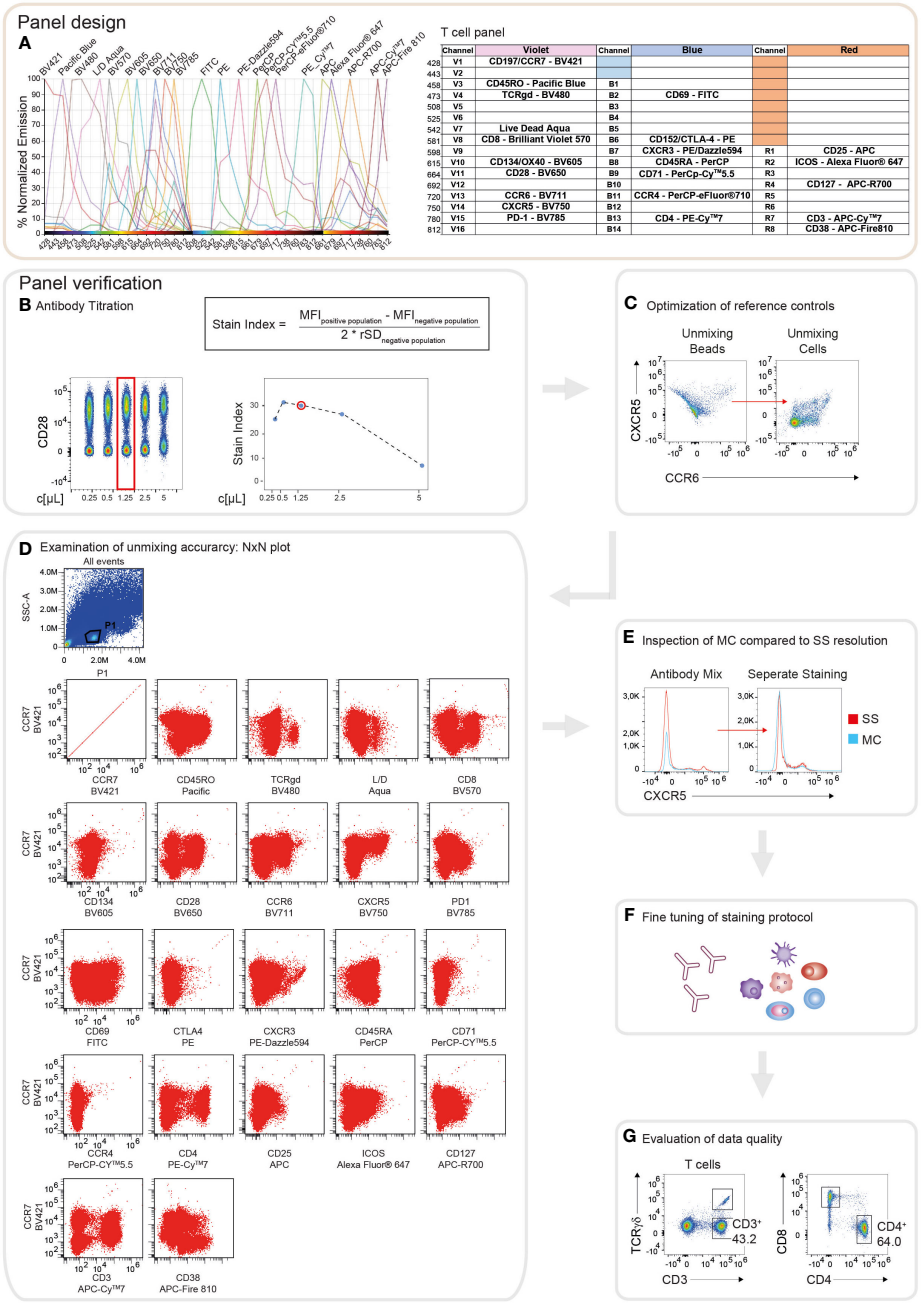
Schematic illustration of the workflow for panel design and verification. **(A)** Theoretical panel design of the T cell immunophenotyping panel. Readout of the Cytek Full Spectrum Viewer (Cytek Biosciences) displaying the spectral signatures of the 22 fluorophores in the 3L configuration of the Cytek Aurora (left). Optical layout of the used markers and fluorophores showing the approximate peak emission wavelengths (nm) (right). **(B)** Overview of the verification strategy for the T cell immunophenotyping panel. Representative example of the antibody titration depicting CD28 conjugated to BV650. Left: Pseudo-color plot of the concatenated files of the 1:2 serial dilutions of CD28. On the x-axis, the respective concentration is shown as µl/test. The red box indicates the final titration result. Right top: The Stain Index (SI) is calculated as the difference between the median fluorescence intensity (MFI) of the positive and negative populations, divided by two times the robust standard deviation (rSD) of the negative population. MFI and rSD were extracted from FlowJo in.csv file format. Right bottom: Diagram depicting the calculated SI of CD28-BV650 over 1:2 serial dilution steps. The x-axis displays the respective concentration as µl/test. The red circle indicates the final titration result. **(C)** Dot plots showing an exemplary illustration of “unmixing” accuracy of CXCR5-BV750 and CCR6-BV711 utilizing single stained compensation beads compared to single stained whole PBMCs. **(D)** NxN plots depicting the quality control of “unmixing” accuracy in the multi-color stained sample, gated on lymphocytes. CCR7-BV421 is shown as a representative example of correct “unmixing”. In every NxN plot, CCR7 is represented on the y-axis and all other fluorophores of the panel on the x-axis. **(E)** Staining resolution of the single stained (SS) tube compared to the fully stained/multi-color (MC) tube on the example of CXCR5-BV750. **(F)** Loss of staining resolution resulted in fine-tuning of the staining protocol, such as separate staining steps and changes in the antibody concentrations. **(G)** Immunophenotyping results were manually gated and compared to published studies to evaluate the quality of the data.

Cell surface markers for the best discrimination of the individual PBMC lineages, as well as activation and inhibitory surface receptors covering the whole PBMC landscape, were selected, following previously published approaches (24, 32, 34, 35, 37). Using a Cytek Aurora with a 3-laser configuration, at the time of panel design, measurements of up to 28 fluorochromes in one panel were possible, using commercially available antibody reagents. Thus, two individual 22-color immunophenotyping panels were generated: one immunophenotyping panel encompassing the majority of PBMC populations, (PBMC panel; **Supplementary Figure 1A**) and one panel for characterizing different Th cell subsets (T cell panel; **Figure 1A**). 19 corresponding markers were included to assess the activation state of the individual subtypes. Possible fluorophores were identified based on the 3-laser configuration of the Cytek Aurora spectral flow cytometer and considering the individual spectra of the fluorescent dyes. Dyes possessing unique peak emissions and a distinct spectral signature, respectively, were selected for the panels. Subsequently, the depicted fluorophores were examined in the “Similarity Matrix” of the SpectroFlo^®^ software, where the “Similarity^TM^ Index” reflects the uniqueness of the fluorophore spectrum for every dye in the panel in comparison to other fluorophores (**Supplementary Figures 2A, B**). The “Similarity^TM^ Index” indicates possible combinations of fluorochromes used in a panel. An index of 0 implies that the spectra are unique, whereas an index of 1 describes identical spectral signatures (**Supplementary Figures 2A, B**). In addition, the algorithm in the SpectroFlo^®^ software determines the “Complexity^TM^ Index”, a value that summarizes all individual “Similarity^TM^ Indices” in the respective panel. This implies that the more dyes bearing high similarities indices exist in the panel, the higher the resulting “Complexity^TM^ Index” is. Thus, the resulting “Complexity^TM^ Index” reflects a measure of the global compatibility of the selected collection of fluorophores. The lower the “Complexity^TM^ index”, the better the individual spectral signatures can be separated, resulting in improved “unmixing” results, decreased spread, and optimal resolution (**Supplementary Figures 2A, B**). A “Complexity^TM^ Index” of 14.6 for the PBMC panel and 13.81 for the T cell panel has been calculated (**Supplementary Figures 2A, B**). Of note, the “Complexity^TM^ Index” just indicates the degree of the complexity to analyze a certain panel, in this case, no specific threshold (only reference values) exists. Key principles for panel establishment in spectral flow cytometry are analogous to conventional flow cytometry and have been detailed reviewed in other studies (32, 40). In brief, markers were categorized into three main groups based on their expression level: Primary markers are highly expressed or characteristic for main cell subsets, thus these markers are assigned to dim fluorophores (e.g., CD45RA in PerCp). Secondary antigens represent receptors possessing intermediate cellular densities or depict differentiation markers for further subset discrimination and are thus allocated to fluorophores with medium brightness (eg. CCR4 in PerCp-eFluor^®^710). Tertiary markers depict proteins expressed at low and unknown densities, respectively, or in populations with low frequencies. Hence, these antigens are assigned to very bright fluorophores (eg. CD25 in APC) (**Figure 1A**). Next, the expression and co-expression of the individual antigens in the different leukocyte populations were assessed. A theoretical panel quality control was performed using the antibody panel grid that shows possible areas of spillover/spread between the different markers (**Figure 1A** and **Supplementary Figure 1A**). In general, the spread is dependent on the expression level of the respective marker on a specific cell type. Antigen assignment was conducted to avoid highly expressed markers being placed in adjacent cells in the same row and/or column as co-expressed markers with low cellular density. Considering the placement of markers within the grid minimizes “unmixing” inaccuracies. Following these guidelines, we were able to successfully design two 22 multi-color spectral flow cytometry panels offering a maximal resolution of all antigens with minimal spread.

### 2.2 Stepwise instruction for successful panel verification

Following the thoughtful design of the panels for the immunophenotyping, their performance regarding antibody sensitivity, antigen separation, and “unmixing” accuracy were evaluated and optimized. First, all antibody reagents were carefully titrated in 2-fold serial dilutions, in line with the manufacturer’s recommendations (for details we refer to the Methods section). Optimal antibody concentrations were determined by a combination of visual assessment of the concatenated files of the distinct dilutions and calculation of the stain index, respectively (**Figure 1B**). Antibody dilutions displaying high separation between the positive and the negative populations, as well as adequate values for the stain index were selected (**Figure 1B**). Next, similar to compensation in conventional flow cytometry, reference controls for qualitative “unmixing” were evaluated applying a combination of single stained compensation beads and PBMCs. Frequently “unmixing” inaccuracies were observed using bead reference controls that could be avoided utilizing single stained cells (**Figure 1C**). In the experiments executed for the presented study, cells were utilized as reference controls for accurate “unmixing”. Due to the low autofluorescence of the cells, the “autofluorescence tag” was not included in the “unmixing” (**Supplementary Figure 1B**). In order to verify the “unmixing” accuracy of the fully stained sample, after the removal of doublets, dead cells, and aggregates, the cleaned data were subjected to NxN permutations (**Figure 1D**). By visual screening of the NxN plots, we were able to assert correct “unmixing” for the majority of the markers. Minor errors were corrected directly in the SpectroFlo^®^ software by carefully aligning the negative and positive populations in the y-dimension. However, the maximum correction did not exceed 1.5%. Subsequently, the impact of “unmixing” on the resolution of the individual makers was verified by comparing the fully stained sample to the single stained tube. In case the fully stained tube (MC) displayed diminished separation of the positive and negative populations compared to the single stained tube (SS), the concentration of the respective antibody was adjusted or the staining protocol was modified (**Figures 1E, F**). For example, the resolution of CXCR5 was elevated by the addition of this antibody in a separate staining step of the protocol (**Figures 1E, F**). After fine-tuning of the staining protocol, generated data were analyzed and evaluated by comparing the obtained results to published studies (**Figure 1G**). In summary, following the described protocol steps for panel design and verification, allows the user to obtain high quality and reproducible flow cytometry data.

### 2.3 Strategies for gating and presentation of PBMC populations and frequencies

**Figure 2:**
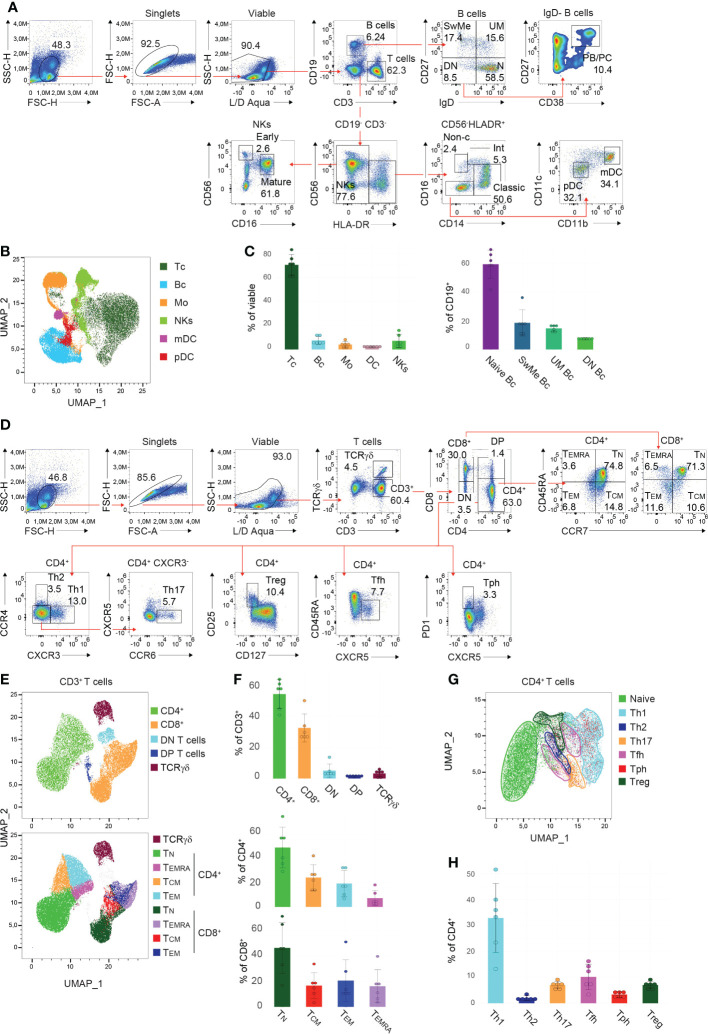
Gating strategy and presentation of PBMC and T cell subsets **(A, D)** Manual gating approaches of the major **(A)** PBMC subsets and **(D)** T cell subsets, respectively, following 24 hours of *in vitro* cultivation. Red arrows depict the relationships across plots. Numbers indicate the percentages of cells in the quadrants or gates. Individual parent gates are referred to on top of the plots when necessary. One representative donor is shown. **(B, E, G)** High-dimensional data analysis using Uniform Manifold Approximation and Projection (UMAP) trained on **(B)** whole PBMCs, **(E)** CD3^+^ T cells, and **(G)** CD4^+^ T cells, respectively, of concatenated donors depicting the accurate separation of the manually gated subsets following 24 hours *in vitro* cultivation. UMAPs were generated in FlowJo. **(C, F, H)** Bar charts illustrating the percentages of indicated subsets within **(C)** viable PBMCs (left), CD19^+^ B cells (right), **(F)** CD3^+^ T cells (top), CD4^+^ and CD8^+^ T cells (bottom), as well as **(H)** CD4^+^ T cells of 6 donors following 24 hours *in vitro* cultivation. Each symbol indicates 1 independent biological sample. Horizontal bars indicate the mean, error bars show the standard deviation. **(A–H)** Data are representative **(A, D)** or show a summary **(B, C, E–H)** of at least 6 independent experiments. DC, dendritic cells; pDC, plasmacytoid DC; mDC, myeloid DC; NK cells, natural killer; Bc, B cells; N, naïve; UM, sunswitched memory; SwMe, witched memory; DN, double-negative; DP, double-positive; PB/PC, plasmablasts/plasma cells; Mo, monocytes; int, intermediate; non-c, non-classical; Tc, T cells; TN, naïve T cells; TCM, central memory T cells; TEMRA, terminally differentiated effector T cells; TEM, effector memory T cells; Th, T helper cells; Treg, regulatory T cells; Tfh, T follicular helper cells; Tph, T peripheral helper cells.

In order to identify the main populations within PBMCs, manual gating was performed (see **Tables 1**, **2** for detailed information regarding the used markers). Following the exclusion of debris, doublets, and dead cells utilizing the scatter profiles and viability dye, respectively, CD3 and CD19 were used to discriminate T cells and B cells (**Figure 2A**). In this context, we could detect on average 70.6% (Interquartile range (IQR) =10.9) of T cells and 7.5% (IQR=5.1) of B cells among viable PBMCs (**Figures 2A, C**). Within the B cell compartment, subsets were further distinguished by CD27 and IgD expression. The majority of B cells, on average 59.4% (IQR=6.6), were classified as naïve B cells (IgD^+^ CD27^-^), followed by unswitched memory (UM) B cells (mean: 14.6%, IQR=3.4, IgD^+^ CD27^+^), switched memory (SwMe) B cells (mean: 18.5%, IQR=5.6, IgD^-^ CD27^+^) and double-negative (DN) B cells (mean: 7.5%, IQR=1.0, IgD^-^ CD27^-^) (**Figures 2A, C**). The IgD^-^ B cell population contains the plasmablasts and plasma cells (PB/PC), respectively, which are characterized as CD38^+^ CD27^+^ and constitute on average 4.1% (IQR=5.8) of IgD^-^ B cells (**Figure 2A**). NK cells were identified within the CD3^-^ CD19^-^ HLA-DR^-^ gate and we observed on average 7.1% (IQR=6.9) of NK cells among viable PBMCs (**Figures 2A, C**). They can be further divided into early and mature NK cells based on the expression of CD56 and CD16 (**Figure 2A**). Monocytes were gated within the CD3^-^ CD19^-^ HLA-DR^+^ compartment, and constituted on average 4.1% (IQR=2.3) among viable PBMCs (**Figures 2A, C**). They could be classified into classical (mean: 43.5%, IQR=11.2, CD14^+^ CD16^-^), intermediate (mean: 5.4%, IQR=4.1, CD14^+^ CD16^+^), and non-classical (mean: 2.1%, IQR=1.0, CD14^-^ CD16^+^) monocytes (**Figure 2A**). DCs were identified within the CD3^-^ CD19^-^ HLA-DR^+^ CD14^-^ CD16^-^ population, and we detected on average 2.1% (IQR=0.6) of DCs among viable PBMCs (**Figures 2A, C**). DCs can be further separated into plasmacytoid DCs (pDCs) (mean: 28.6%, IQR=13.7, CD11b^low^ CD11c^low^) and myeloid DC (mDCs) (mean: 45.3%, IQR=8.2, CD11b^+^ CD11c^high^) (**Figure 2A**). Furthermore, Uniform Manifold Approximation and Projection (UMAP) was performed to arrange phenotypically similar events into distinct clusters for presenting similarities and differences inside each population and for interior comparison of the different populations (**Figure 2B**). This dimensionality reduction analysis using the lineage discrimination parameters in the PBMC population highlighted the distinct separation of the individual subsets when manually gated clusters were projected on the UMAP (**Figure 2B**). The results of these projections emphasize not only the power of visualization approaches but also highlight the quality of our panel design and manual gating approaches.

**Table 1:** Descriptive summary of PBMC panel.

Marker	Fluorophore	Alternative name	Purpose
**CD126**	BV421	IL-6R1	Activation marker involved in stimulation of B and T cells, expressed on plasma cells, activated B cells, T cells, monocytes
**HLA-DR**	eFlour450		Activation marker expressed on B cells, T cells, APCs; marker for monocyte and DC lineage
**IgD**	BV480		B cell differentiation
**Live/Dead**	L/D Aqua		
**CD16**	BV570		Monocyte and NK cell differentiation
**IgG**	BV605		B cell differentiation
**CD4**	cFluorV610		CD4 T cell lineage
**CD56**	BV650	Neural cell adhesion molecule (NCAM)	Pan NK cells
**CD95**	BV711	Fas	Activation marker; involved in apoptosis, expressed on T cells, B cells, monocytes
**CD11b**	BV750	Integrin alpha M	DC phenotyping
**CD279**	BV785	Programmed cell death protein 1 (PD-1)	Inhibitory receptor expressed on activated T cells, B cells and myeloid cells
**CD11c**	BB515	Integrin alpha X	DC lineage marker
**CD69**	FITC		Early activation marker expressed on leukocytes
**CD27**	PE		T and B cell differentiation, expressed on T cells, B cells, NK cells
**CD70**	PE-Dazzle594	CD27 ligand	Activation marker expressed on T cells, B cells, NK cells, pDCs
**CD14**	PerCp-Cy5.5		Monocyte differentiation
**CD3**	PE-Cy7		Pan T cells
**CD25**	APC	IL-2R α chain	Treg marker; activation marker expressed on T cells, B cells, monocytes/macrophages
**CD169**	Alexa Flour647	Siglec-1	DCs, macrophages, involved in cell interaction
**CD86**	BD Horizon APC-R700		Activation marker on B cells, T cells, monocytes/macrophages, DCs; co-stimulation of T cell activation
**CD19**	APC-Cy7		B cell lineage
**CD38**	APC-Fire810	Cyclic ADP ribose hydrolase	Monocyte, DC, T cells, B cells and NK cell activation/differentiation

**Table 2:** Descriptive summary of T cell panel.

Marker	Fluorophore	Alternative name	Purpose
**CD197**	BV421	CCR7	T cell differentiation
**CD45RO**	Pacific Blue		T cell differentiation, activated and memory T cell marker
**TCRγδ**	BD Horizon 480		Pan γδ T cell
**Live/Dead**	L/D Aqua		
**CD8a**	BV570		CD8 T cell lineage
**CD134**	BV605	OX40	Activation marker expressed on T cell subsets
**CD28**	BV650		T cell co-stimulation molecule
**CCR6**	BV711		Th17 lineage marker
**CXCR5**	BV750		Tfh lineage marker
**CD279**	BV785	Programmed cell death protein 1 (PD-1)	T cell inhibitory receptor
**CD69**	FITC		Early activation marker
**CD152**	PE	Cytotoxic T-lymphocyte-associated Protein 4 (CTLA-4)	T cell inhibitory receptor
**CXCR3**	PE-Dazzle594		Th1 lineage marker
**CD45RA**	PerCP		T cell differentiation, marker for naïve and effector T cells
**CD71**	PerCP-Cy5.5		Transferrin receptor; marker for proliferating cells
**CCR4**	PerCP-eFlour710		Th2 lineage marker
**CD4**	PE-Cy7		CD4 lineage marker
**CD25**	APC	IL-2R α chain	Treg marker; activation marker
**CD278**	Alexa Fluor 647	Inducible T-cell COStimulator (ICOS)	Activation marker, co-stimulation of T cell proliferation
**CD127**	BD Horizon APC-R700	IL-7Rα	T cell differentiation
**CD3**	APC-Cy7		Pan T cells
**CD38**	APC-Fire810	Cyclic ADP ribose hydrolase	Activation marker

Due to the complexity of the T cell compartment, a separate staining panel and gating strategy was established. After the exclusion of debris, doublets, and dead cells, T cells were classified into TCRγδ and CD3^+^ (TCRαβ) cells (**Figure 2D**). The CD3^+^ population was then further divided into CD4^+^ and CD8^+^ T cells, as well as double-negative and double-positive (DP) T cells (**Figure 2D**). In summary, we detected on average 54.5% (IQR=12.7) of CD4^+^ T cells, 32.6% (IQR=5.1) of CD8^+^ T cells, 5.1% (IQR=1.8) of DN T cells, 1.3% (IQR=0.4) of DP T cells, and 3.2% (IQR=3.2) of TCRγδ T cells (**Figures 2D, F**). CD45RA and CCR7 within the CD4^+^ and CD8^+^ T cell gates allow for the separation of cells into the different effector/memory states (**Figure 2D**). Thus the following populations were identified within CD4^+^ T cells: naïve (TN) (mean: 49.1%, IQR=20.3, CCR7^+^ CD45RA^+^), central memory (TCM) (mean: 24.4%, IQR=10.7, CCR7^+^ CD45RA^-^), effector memory (TEM) (mean: 19.3%, IQR=16.9, CCR7^-^ CD45RA^-^) and terminally differentiated effector (TEMRA) (mean: 7.2%, IQR=5.8, CCR7^-^ CD45RA^+^) CD4^+^ T cells; as well as within the CD8^+^ T cells: naïve (TN) (mean: 46.5%, IQR=24.3, CCR7^+^ CD45RA^+^), central memory (TCM) (mean: 16.7%, IQR=7.6, CCR7^+^ CD45RA^-^), effector memory (TEM) (mean: 20.7%, IQR=13.9, CCR7^-^ CD45RA^-^) and terminally differentiated effector (TEMRA) (mean: 16.2%, IQR=10.9, CCR7^-^ CD45RA^+^) CD8^+^ T cells (**Figures 2D, F**). However, the individual donors showed a higher degree of biological variance in the T cell effector states. In addition, dimensionality reduction was trained on CD3^+^ T cells, and applying the manually gated populations on the generated UMAP resulted in distinct clusters of the T cell subsets described above (**Figure 2E**). Further, within the CD3^+^ CD4^+^ population, the different Th cell subsets were classified as follows: Th1 (mean: 32.9%, IQR=13.1, CCR4^-^ CXCR3^+^), Th2 (mean: 2.0%, IQR=1.2, CCR4^+^ CXCR3^-^), Th17 (mean: 7.0%, IQR=1.2, CCR4^-^ CXCR3^-^ CXCR5^-^ CCR6^+^), regulatory T cells (Treg) (mean: 7.0%, IQR=1.2, CD127^low^ CD25^+^), T follicular helper (Tfh) (mean: 10.1%, IQR=6.6, CD45RA^-^ CXCR5^+^) and T peripheral helper (Tph) (mean: 3.4%, IQR=1.4, CXCR5^-^ PD-1^high^) cells (**Figures 2D, H**). Again high-dimensionality data analysis of CD3^+^ CD4^+^ PBMCs generated a UMAP depicting accurately the manually gated CD4^+^ effector/memory states (**Supplementary Figure 4A**) and the individual Th cell subsets (**Figure 2G**) except for Tph cells that are scattered within the Th2, Th1 and Treg clusters. The described gating hierarchy is following published reports (24, 32, 34, 35, 37), and demonstrates a state-of-the-art strategy for the identification of the major PBMC subsets.

### 2.4 Multi-color flow cytometry allows to detect stable PBMC subset distribution upon Phytohaemagglutinin (PHA) stimulation

Thawed PBMCs from 6 HCs were cultured in the presence/absence of PHA for 24 hours and subsequently subjected to immunophenotyping (**Supplementary Figure 3A**). After the exclusion of debris, doublets, and apoptotic cells by using viability dye staining, all main PBMC subsets, in detail B cells, T cells, monocytes, (DCs), and NK cells were detectable. The frequencies within the PBMC subtypes between the subjects differed slightly due to biological variance (**Supplementary Figure 3B**). Activation by PHA did not induce significant changes in the cell composition of the PBMC compartments within the individual donors, indicating that PHA is not inducing apoptosis of a specific cell subset (**Supplementary Figure 3B**, top). Similar tendencies were observed within the B cell compartment, displaying unaltered proportions of DN, switched memory, unswitched memory, and naïve B cells between non-activated and activated states (**Supplementary Figure 3B**, bottom). Investigating the effector states among T cells, we observed unaltered frequencies of CD4^+^ and CD8^+^ naïve and effector (central memory, terminally differentiated effector, effector memory) T cells upon PHA stimulation (**Supplementary Figure 3C**). Remarkably, the individual donors showed variability in the individual ratios of naïve to effector T cells (**Supplementary Figure 3C**). Th cell subsets were defined by the expression of surface markers, as described in **Figure 2D**. Overall, the detected percentages are comparable to the existing literature (24), showing a high abundance of Th1 cells and lower frequencies of Th2 and Tph cells. Upon activation with PHA, only slight differences in the percentages of Th subsets among CD4^+^ T cells were assessed.

All in all, by utilizing these immunophenotyping panels we could demonstrate that PHA did not alter the distribution of the individual subsets nor induced subtype-specific apoptosis.

### 2.5 Activating and inhibitory receptors are differentially expressed in individual PBMC compartments and are modulated by PHA stimulation

Heatmaps are a valid tool to globally visualize alterations in the activation marker landscape of immune cells. Thus, we calculated heatmaps using the median fluorescent intensity (MFI) of all included surface markers for the respective subsets. First, we observed a subset-specific distribution of activating and inhibitory receptor expression. CD27 is highly expressed in SwMe and UM B cells (**Figure 2A**). Accordingly, B cells display the highest baseline levels compared to other subsets (**Supplementary Figure 3D**). The ligand of CD27, CD70, is most abundant in pDCs (**Supplementary Figure 3D**). In line with the literature, CD27 and CD70 were not detected in monocytes (**Supplementary Figure 3D**). In general, monocytes show elevated expression of several markers, including CD169, CD69, PD-1, CD126, CD95, and CD86 in the non-stimulated condition, that were further induced by PHA activation (**Supplementary Figures 3D, F, G**). Furthermore, monocytes upregulated CD38, HLA-DR, and CD25. Overall, we detected a strong induction of CD69 among all PBMC subsets upon stimulation with PHA reflecting a global activation (**Supplementary Figures 3D, F**). In contrast, CD27 and CD70 are down-regulated in B cells and NK cells upon PHA treatment.

Within the T cell compartment, naïve CD4^+^ and CD8^+^ T cells exhibit the highest abundance. Accordingly, high levels of CD45RA and low expression of CD45RO were illustrated in the heatmap (**Supplementary Figure 3E**). Furthermore, we detected the highest levels of Inducible T-cell costimulatory (ICOS) in Th2 cells in response to PHA (**Supplementary Figures 3E, I**). As Tph cells are defined by high Programmed cell death protein 1 (PD-1) expression, elevated expression of this molecule compared to other subsets was observed that was also induced upon activation (**Supplementary Figures 3E, I**). Interestingly, Tph cells display an elevated activation phenotype, characterized by strong upregulation of CD134 (OX-40) and CD25 in the stimulated condition. In addition, CD25 is an indispensable molecule for the characterization and function of Tregs, accordingly high levels were identified in this subset in the non-stimulated condition, as well as upon PHA treatment (**Supplementary Figure 3E**). Overall, CD69 and CD38 were strongly enhanced in all T cell subsets in the activated state compared to the baseline (**Supplementary Figure 3E**). CCR7, being responsible for the afferent trafficking of leukocytes to lymph nodes, was upregulated in response to PHA. Interestingly, the inhibitory receptor CTLA-4 was solely weakly expressed in Tregs, when compared to Th1 cells and total CD4^+^ T cells (**Supplementary Figure 4B**). CTLA-4 was up-regulated in the majority of T cell compartments upon stimulation (**Supplementary Figure 3E**).

In summary, we demonstrate that PHA constitutes an adequate method for the stimulation of PBMCs. Furthermore, the established panels allow to detect an overview of the activation status of different leukocyte subtypes induced by PHA.

### 2.6 Advanced immunophenotyping of PBMCs allows to detect drug-induced effects on cell activation

**Figure 3:**
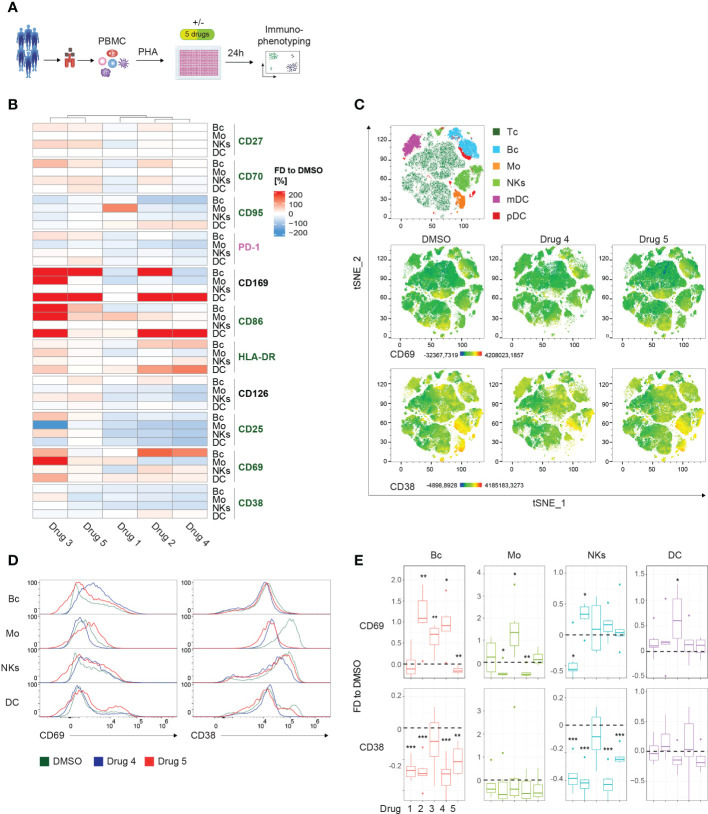
Drug screening approach for whole PBMCs. **(A)** Schematic illustration of the workflow. PBMCs of 6 healthy controls (HCs) were PHA-stimulated and cultured for 24 hours in the presence of 5 immunomodulatory drugs and DMSO as control, respectively. **(B)** Summary heatmap depicting fractional differences (FD) of the marker expression as median fluorescent intensity (MFI) of drug-treated PBMCs compared to DMSO control in the indicated PBMC subsets of 6 pooled HCs. Each column represents the individual drugs (drugs 1-5), and rows show the marker expression in the distinct PBMC compartments. Heatmaps were generated in R using the “Complex Heatmap” package; data are represented as the FD in percentages of the marker MFI of the individual compounds compared to DMSO and clustered by column. DMSO control was set to 0. Outliers (x<-150%, x>150%) were removed from the matrix. The color code depicts activation (green), inhibitory (purple), and lineage-specific (black) markers. **(C)** High-dimensional data analysis on viable PBMCs from concatenated HCs using the t-Stochastic Neighbor Embedding plot (tSNE) plugin in FlowJo displaying manually gated clusters of the respective PBMC subsets (first row) and density plots illustrating the global marker expression of CD69 and CD38, respectively, in the DMSO condition and in response to treatment with drug 4 and drug 5 (second and third row). Yellow-orange colors depict areas of high marker expression, whereas dark green-blue areas indicate areas of lower marker expression. **(D)** Histograms showing CD69 and CD38 expression, respectively, in the indicated PBMC subsets. Colors illustrate the treatment condition, as DMSO is green, drug 4 treatment is blue and drug 5 treatment is red. **(E)** Summary box plots showing the FD of the marker MFI of drug 1-5 compared to DSMO in the indicated PBMC subsets. The intercept line equates to DMSO control. (*P < 0.05, **P < 0.01, and ***P < 0.001). **(A–E)** The DC subset encompasses pDCs and mDCs. Data are representative **(D)** or show a summary **(B, C, E)** of at least 6 independent experiments. DC, dendritic cells; pDC, plasmacytoid DC; mDC, myeloid DC; Mo, monocytes; NKs, natural killer cells; Bc, B cells.

To demonstrate the power of our approach for drug screening, we activated PBMCs in the presence of different immunomodulating compounds for 24 hours (**Figure 3A**). Following immunophenotyping, changes in the median fluorescent intensity (MFI) compared to DMSO for the individual markers of every subset were extracted. To interrogate and visualize specific activating/inhibitory signatures of the compounds and the corresponding relation to the individual subsets, we generated a heatmap plot. Hierarchical clustering revealed 2 groups possessing similar activation signatures: cluster 1 (drugs 3 and 5) demonstrated a rather mixed or even activation-promoting phenotype, while the second cluster (drugs 1, 2 and 4) displayed a tendency for an inhibitory mode-of-action (**Figure 3B**). Of note, we observed a strong induction of CD169 in DCs upon treatment when compared to DMSO. Interestingly, in B cells upregulation of CD169 was observed in response to drugs 2, 3, and 5 (**Figure 3B**). Of note, the modulation of CD25, PD-1, and CD126 appeared to be more inconsistent when comparing the different conditions. In general, CD69 was upregulated in the majority of PBMC compartments after culturing in the presence of different drugs. Contrary, CD38 displayed reduced expression levels in response to the various tested compounds. To highlight the different potential of individual drugs on these markers, we selected one compound per the above-mentioned clusters and generated dimensionality reduction approaches using tSNE, comparative histograms, and statistical analysis (**Figures 3C–E**). First, we performed dimensionality reduction of the dataset using tSNE, and then explored the generated plot by 3^rd^ parameter color mapping (**Figure 3C**). This t-SNE map allowed us to illustrate the different PBMC compartments, as well as the drug-induced modulation of the expression of two selected receptors, namely CD69 and CD38. Although the tSNE plots show a global picture of the marker expression (**Figure 3C**), a comprehensive analysis of individual alterations in the activation marker profile is difficult to extract. In order to enable a quantitative assessment, manual validation utilizing approved flow cytometry analysis tools and statistical validation were performed. In the corresponding histograms, the downregulation of CD38 in monocytes when treated with drugs 4 and 5 could be demonstrated (**Figure 3D**). In response to drug 4, CD69 tended to be downregulated in monocytes but was induced in B cells (**Figure 3D**). Statistical analysis showed significant down-modulation of CD38 in response to drug 4 and drug 5 in the majority of analyzed PBMC compartments when compared to the DMSO-treated control. Of note, the overall distribution of the individual cell subsets was not significantly impaired (**Supplementary Figure 4C**).

In summary, we detected individual signatures, highlighting the usability of the presented method to assess compound-induced effects. Furthermore, we show state-of-the-art techniques to enable fast and clear visual and statistical validation of the observed modes-of-action.

### 2.7 Alterations in expression profiles define drug-induced effects in Th-cell subsets

**Figure 4:**
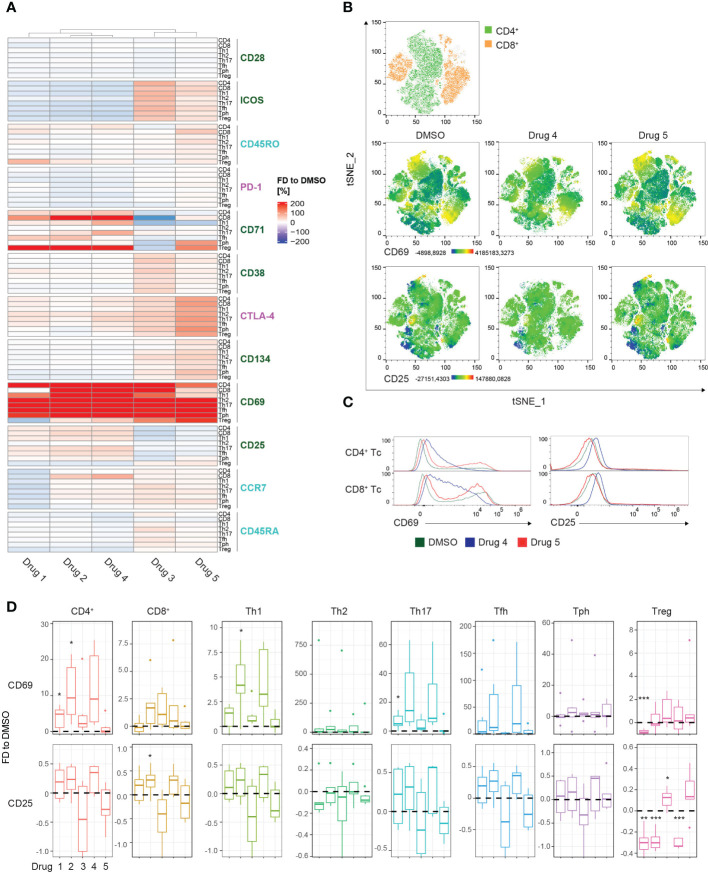
Drug screening approach for T cell subsets. **(A)** Summary heatmap depicting fractional differences (FD) of the marker expression as median fluorescent intensity (MFI) of drug-treated PBMCs compared to DMSO control in the indicated T cell/Th cell subsets of 6 pooled HCs. Each column represents the individual drugs (drugs 1-5), and rows show the marker expression in the distinct PBMC compartments. Heatmaps were generated in R using the “Complex Heatmap” package; data are represented as the FD in percentages of the marker MFI of the individual compounds compared to DMSO and clustered by column. DMSO control was set to 0. Outliers (x<-150%, x>150%) were removed from the matrix. The color code depicts activation (green), inhibitory (purple), and maturation (turquoise) markers. **(B)** High-dimensional data analysis on CD3^+^ T cells of PBMCs from concatenated HCs using the t-Stochastic Neighbor Embedding plot (tSNE) plugin in FlowJo displaying manually gated clusters of the respective T cell subsets (first row) and density plots illustrating the global marker expression of CD69 and CD25, respectively, in the DMSO condition and in response to treatment with drug 4 and drug 5 (second and third row). Yellow-orange colors depict areas of high marker expression, whereas dark green-blue areas indicate areas of lower marker expression. **(C)** Histograms showing CD69 and CD25 expression, respectively, in the indicated T cell/Th cell subsets. Colors illustrate the treatment condition, as DMSO is green, drug 4 treatment is blue and drug 5 treatment is red. **(D)** Summary box plots showing the FD of the marker MFI of drugs 1-5 compared to DSMO in the indicated T cell/Th cell subsets. The intercept line equates to DMSO control. (*P < 0.05, **P < 0.01, and ***P < 0.001) **(A–D)** Data are representative **(C)** or show a summary **(A, B, D)** of at least 6 independent experiments. Th, T helper; Treg, regulatory T cells; Tfh, T follicular helper and Tph, T peripheral helper.

To compare differential marker expression across all subsets, the MFIs of the respective T cell populations were extracted and visualized in a heatmap (**Figure 4A**). Hierarchical clustering uncovered 2 distinct clusters, group 1 contained drugs 1, 2, and 4, whereas group 2 included drugs 3 and 5. However, complementary to the previous heatmap, no obvious activation pattern distinguished the groups. The strongest induction was observed for CD69 expression among all T cell subsets in both clusters. Admittedly, the extreme effects on CD69 and the resulting visual scaling of the heatmap might mask the modulation of other markers (**Figure 4A**). A strategy to avoid these scaling effects would include the individual visualization and scaling of subgroups dependent on the requirements of the analysis. Interestingly, treatment with drug 3 induced decreased expression of CD71 compared to DMSO, showing the most substantial effect on CD8^+^ T cells. In contrast, treatment with drugs 1, 2, and 4 resulted in a strong upregulation of CD71 in CD8^+^ T cells, and also, to a slighter degree, in the CD4^+^ T cell subsets. This highlights the potential of the presented method to detect selective drug-specific effects (**Figure 4A**). High-dimensional analysis using t-SNE generated a global landscape of the expression patterns of CD69 and CD25 (**Figure 4B**). As an additive analysis tool, drugs from the 2 different clusters were selected for visualization in the 3^rd^ parameter color-mapped plots. Although the plots highlighted drug-induced differences in the marker expression profile in the peripheral CD4^+^ and CD8^+^ T cells, further investigation of specific subsets remained elusive (**Figure 4B**). To fill this gap, comparative histograms allowed detailed analysis of distinct expression signatures, and box plots enable statistical analysis of the MFIs (**Figures 4C, D**). In line with results extracted from the heatmap, drug 4 induced upregulation of CD69 and CD25, whereas drug 5 showed only moderate effects on CD69 expression and downregulated CD25 in CD4^+^ and CD8^+^ T cells (**Figures 4C, D**). Similarly, drug 4 and drug 5 caused differential regulation of CD25 in the different Th cell subsets (**Figures 4A, C, D**). Of note, the global distribution of the particular T cell subsets was not altered by drug treatment (**Supplementary Figure 4D**). Together, these data demonstrate the power of the presented approach to elucidate drug-specific effects in different Th cell subsets.

### 2.8 Immunophenotyping exposes disease-specific expression marker profiles in RA patients

**Figure 5:**
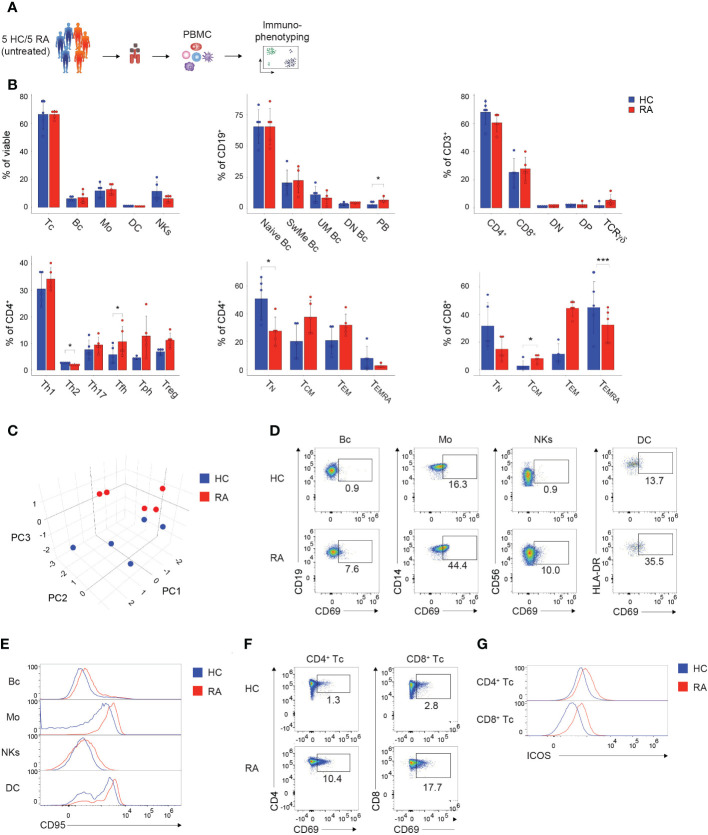
Immunophenotyping of patients suffering from autoimmune diseases. **(A)** Schematic illustration of the workflow. Frozen PBMCs of 5 naïve, untreated, female RA patients and 5 sex-matched and age-matched HCs were thawed and directly subjected to immunophenotyping using the established PBMC and T cell panel. **(B)** Bar charts depicting the percentages of the indicated subsets within viable PBMCs (left, top), CD19^+^ B cells (middle, top), CD3^+^ T cells (right, top), CD4^+^ T cells (left, bottom), CD4^+^ T cells (middle, bottom), and CD8^+^ T cells (right, bottom). Each symbol indicates 1 independent biological sample. Statistical comparisons were done by Student’s t-test, comparing the 5 individual replicates of RA patients to HC. Significance was defined as p-value (*P < 0.05 and ***P < 0.001). **(C)** 3-dimensional PCA depicting the combined activation marker expression levels resulting from the immunophenotyping using the PBMC and T cell panel. Each symbol indicates 1 independent biological sample. Blue dots show HCs and red dots illustrate RA patients. **(D, F)** Spectral flow cytometry analysis showing CD69 expression in the indicated **(D)** PBMC and **(F)** T cell subsets of 1 representative HC and RA patient, respectively. Numbers indicate the percentage of cells in the quadrants or gates. **(E, G)** Histograms showing **(E)** CD95 expression or **(G)** ICOS expression in the indicated **(E)** PBMC and **(G)** T cell subsets of 1 representative HC and RA patient, respectively. **(B–E)** The DC subset encompasses pDCs and mDCs. Data are representative **(D–G)** or show a summary **(B, C)** of at least 3 independent experiments. DC, dendritic cells; Mos, monocytes; NKs, natural killer cells; Bc, B cells; Tc, T cells; N, naïve; UnMe, unswitched memory; SwMe, switched memory; DN, double-negative; DP, double-positive; T_N_, naïve T cells; T_CM_, central memory T cells; T_EMRA_, terminally differentiated effector T cells; T_EM_, effector memory T cells; Th, T helper cells; Treg, regulatory T cells; Tfh, T follicular helper cells; Tph, T peripheral helper cells; RA, Rheumatoid arthritis; HC, healthy control; PCA, Principal Component Analysis.

In order to demonstrate the applicability of the presented immunophenotyping panels for the screening and diagnosis of patients, we analyzed 5 naïve, untreated RA patients and 5 age-matched and sex-matched HCs using the established spectral flow cytometry approach (**Figure 5A**). First, alterations in the PBMC subset composition between RA patients and HCs were assessed. RA patients displayed no significant changes in the major PBMC subtypes, such as T cells, B cells, monocytes, DCs, and NK cells, but showed elevated levels of plasmablasts and TCRγδ T cells compared to HCs (**Figure 5B**). Within the Th cell compartment, RA patients displayed decreased percentages of Th2 cells, while levels of Tfh, Tph, and Treg cells were enhanced compared to HCs (**Figure 5B**). Furthermore, naïve and T_EMRA_ CD4^+^ and CD8^+^ T cells were reduced in RA patients (**Figure 5B**). 3-dimensional Principal Component Analysis (PCA) of the combined datasets of activation and inhibitory marker expression displayed a clear separation of the RA cohort from the HC, suggesting a different immunological activation profile in patients suffering from autoimmune diseases (**Figure 5C** and **Supplementary Figure 5C**). Overall, RA patients displayed an elevated expression of several activation markers compared to HCs (**Supplementary Figures 5A, B**), corresponding to the inflammatory immune status in individuals suffering from autoimmune diseases. For instance, increased percentages of CD69 were detected in B cells, monocytes, NK cells, DCs, as well as CD4^+^ and CD8^+^ T cells in the RA cohort (**Figures 5D, F**; **Supplementary Figures 5A, B**). Further, CD95 expression was enhanced in B cells, monocytes, NK cells and DCs of RA patients (**Figure 5E**). CD4^+^ and CD8^+^ T cells expressed higher percentages of ICOS in the RA cohort, while the naïve marker molecule CD45RA was decreased compared to HCs (**Figure 5G**; **Supplementary Figure 5B**).

In summary, the presented immunophenotyping approach allows to characterize and identify individuals suffering from autoimmune diseases, such as RA, and can thus serve as a potential tool for the diagnosis of patients.

## 3 Discussion

In the presented study, we established an *in vitro* drug screening assay based on readouts from spectral flow cytometry, which offers the possibility to simultaneous measure more than 20 fluorochromes using a 3-laser configuration. Two 22-color immunophenotyping panels thereby enabled an in-depth characterization of B cells, monocytes, DCs, and NK cells and one panel examined T cells, their effector/memory subsets, as well as the different Th cell compartments. Further, 19 different activation and inhibitory markers outlining the whole PBMC landscape were included in the panels. The established system allowed to define differences between HCs and RA patients, suggesting a potential use of this assay for the identification of patients with autoimmune diseases. In addition, the spectral flow cytometry approach was advanced by combining immunophenotyping with an *in vitro* screening assay for immunomodulatory drugs detecting alterations in cellular compositions and activation profiles in PBMCs.

Although whole blood might be technically easier to obtain and constitutes a potentially useful approach to identify certain disease entities, PBMCs possess several additional advantages and serve as a convenient tool to assess the effects of compound treatment. For immunophenotyping assays frozen PBMCs enable the constant availability of sex-matched and age-matched samples, thus reducing variability between donors and experiments. In addition, PBMCs provide the possibility for more complex functional studies, as they can be utilized to detect pathogenic profiles in patients with autoimmune diseases (24), as well as to investigate drug-induced effects by high-content microscopy (39).

To identify drug-induced changes in the expression profile of activation and inhibitory receptors, PBMCs were *in vitro* stimulated. PHA was utilized as an activation stimulant, since it adequately stimulates different immune cells, including T cells (41–43). In the presented study, we titrated the concentration of PHA to exclude apoptosis and to adequately activate leukocytes. We observed high levels of viable cells (around 80% of total PBMCs), as well as no difference in PBMC subset distribution upon 24 hours of PHA activation, excluding apoptosis of one specific compartment. Furthermore, we detected stable activation in all PBMC subsets, indicated by the upregulation of several markers in response to PHA treatment. These results demonstrate that PHA efficiently stimulates human leukocytes, and in particular Th cells. Specific T cell lineages are characterized by the expression of activation markers, as Tregs are defined by the expression of CD25 and Tph cells can be distinguished from other subsets by expressing high levels of PD-1 and ICOS (44). This raises concerns in the identification of these subsets. However, we could not observe significant alterations in the distribution of the Th cell subsets comparing prior and post 24 hours activation with PHA. Thus, the presented approach allows us to identify and characterize the distinct Th cell subsets irrespective of the used PHA stimulation.

The appropriate detection and activation of the different Th cell lineages are pivotal for studies elucidating the effects of immunomodulatory drugs since they are dysregulated in various autoimmune disorders and contribute to the pathogenesis of these diseases (8, 11, 12). In this context, particularly Th1, Th17, and Tfh cells have been linked to the pathophysiology of autoimmune conditions, such as RA, SLE, and MS; whereas Tregs and Th2 cells are considered to exert immune-suppressive functions (8, 11, 12, 14). In the presented study we detected decreased proportions of Th2 and increased levels of Tfh, Tph and Tregs in RA patients compared to HCs, corresponding to current reports (8, 13–15). However, further insight into the activation profile of immune cells is essential for a more detailed characterization of patients suffering from autoimmune diseases, as well as for the screening of immunomodulatory drugs (24).

In order to assess the activation status of the immune cell subsets, several activation markers covering the whole range of the immune landscape were included. CD69 and CD25 are broadly expressed and rapidly induced following activation of leukocytes, thus representing state-of-the-art activation markers used in (spectral) flow cytometry (45–48). Indeed, we were able to confirm the excellent performance of these markers in immunophenotyping of patients, as well as in an *in vitro* drug screening approach. Contrary to CD69 and CD25, which were induced in the majority of assessed populations, other markers exhibit more specific expression patterns in the presented study. Elevated expression levels of HLA-DR were detected on monocytes, as well as B cells. HLA-DR is a member of MHC class II molecules and is known to be expressed on B cells, activated T cells, and APCs (49–51). HLA-DR molecules are upregulated in response to mitogenic or antigenic stimulation (52, 53). In response to PHA, HLA-DR is strongly induced in monocytes, but not in CD56^+^ NK cells. In general, HLA-DR expression on NK cells is primarily considered a late activation marker, and so far, it is not clear whether HLA-DR expression is linked to NK cell differentiation or can be induced by PHA (54, 55). Similarly, CD169, also known as Siglec-1, is solely expressed on macrophages and dendritic cells in response to type I interferon-signaling (56, 57) and mediates cell-cell adhesion (58). Surprisingly, in the presented study, CD169 was expressed in monocytes in response to treatment with PHA. Considering that PHA binds to sugars on glycosylated surface proteins and crosslinks them, one might speculate that PHA can bind directly to receptors of type I interferon-signaling or to certain pattern recognition receptors (PRRs). Monocytes/macrophages and DCs, secrete type I interferons in response to PRR-mediated signaling (59–61). This might explain an indirect mechanism of CD169 induction in monocytes by PHA. However, further studies are warranted to investigate the effect of PHA on the upregulation of CD169 in monocytes. The different T cell subsets were characterized by stable expression of CD28, whereas particularly strong upregulation of ICOS in Th2; CD38 in CD8^+^, Th1, Th2, Treg and Tph; and CD134 (OX40) in Treg and Tph was induced by PHA stimulation. Similar patterns were observed in PBMCs of RA patients, as ICOS, CD134 and CD71 were elevated in several Th cell subsets of RA patients compared to HCs. Interestingly, at steady-state CTLA-4 was higher expressed in Th1 and total CD4^+^ T cells compared to Tregs, which is in line with published datasets (24). Notably, in the presented study the CTLA-4 staining was applied solely extracellularly compared to reports describing additional intracellular staining, which might also influence the levels of detectable CTLA-4 (62).

To demonstrate the applicability of the presented methodology for *in vitro* drug screening, we investigated the effects of 5 different immunomodulatory compounds. In general, the results obtained by such immunophenotyping studies constitute complex multi-parameter datasets containing a variety of different variables and thus require high-dimensional computational analysis. In the presented study, we show state-of-the-art techniques to enable fast and clear visual and statistical validation of the measured alterations. Heatmaps have emerged as a compelling tool to visualize global changes in the expression patterns of different populations. Throughout our analysis, heatmaps and hierarchical clustering provided an overview of drug-induced effects on the distinct PBMC subsets. However, there are limitations in the interpretation of heatmaps, since data scaling of extreme outliers can create a visual bias, resulting in a misleading understanding of the results. Thus, heatmaps should be complemented by quantitative and statistical analysis (63). We confirmed the results from the heatmap analysis by including comparative histograms of the MFIs and statistical analysis using box plots and one-sample t-tests. Considering the experimental setup of this study combining flow cytometry-based drug screening and bioinformatics downstream analysis, effortless upscaling to high-throughput concepts can be achieved. Computational automation of multi-parameter screening datasets can be performed by applying R-based toolkits combing quality control, visualization methods, and preliminary algorithm-based analysis (64).

In summary, we have established a robust and reproducible state-of-the-art spectral flow cytometry approach and an *in vitro* drug screening tool. Advanced immunophenotyping constitutes a novel combination of flow cytometry-based characterization of PBMCs, functional drug testing by detecting dynamics in the cellular activation profile, and comprehensive computational analysis. Further, this study provides detailed information on spectral flow cytometry panel design, gating strategies, percentages of individual PBMC subsets, and potential application spectra of immunophenotyping. Based on the possibility to detect cellular alterations in autoimmune diseases, as well as drug-induced effects, advanced immunophenotyping might constitute a promising strategy for personalized treatment approaches in autoimmune diseases and beyond.

## 4 Methods

### 4.1 Human subjects and ethical aspects

Blood samples from 6 female healthy individuals were collected at the Division of Rheumatology at the Medical University of Vienna. Healthy donors are age-matched (35 years ± 3.1), with no drugs at the time of collection and no laboratory detectable parameters indicating an autoimmune disease. For immunophenotyping of patients, blood samples from 5 naïve, untreated, female RA patients (50 years ± 10.4) and 5 female age-matched healthy donors were collected. Written informed consent was obtained from all participants (age ≥18 years) according to the Declaration of Helsinki. Ethical approval for this study was granted by the ethics committee of the Medical University of Vienna, Austria (2071/2020; 1073/2021; 1075/2021; 1448/2019). All research was conducted in compliance with fundamental ethical principles, as stated in the Charter of Fundamental Rights of the European Union (2010/C/83/2), in agreement with Horizon 2020 Ethics guidelines.

### 4.2 PBMC isolation and freezing

Blood was collected into blood collection tubes containing heparin. Mononuclear cells were isolated from whole blood utilizing Pancoll density gradient centrifugation. Briefly, 10 mL of whole blood were diluted at a ratio of 1:1 with PBS, carefully added on top of 15 ml Pancoll (PAN-Biotech) solution, and centrifuged at 530 g for 22 minutes at room temperature (RT) without break. After centrifugation, a clearly separated white layer containing PBMCs was carefully transferred in a new 50 mL Falcon tube, washed with PBS, and centrifuged at 400 g for 8 min at 4°C. Cell pellets were resuspended in 5 mL PBS. Human PBMCs were counted at a 7 – 15 µm diameter range utilizing a Z2 Coulter Particle Count and Size Analyzer (Beckman Coulter). Following a spin (400 g, 8 min, 4°C), PBMCs were resuspended at a concentration of 10 – 20 x 10^6^ cells/mL with freezing medium (RPMI-1640 supplemented with 20% FCS (Gibco) and 15% DMSO (Sigma)) in cryovials and stored in a CoolCell FTS30 cell freezing container for 24 hours. Afterward, cryovials were transferred to liquid nitrogen for long-term storage.

### 4.3 Thawing of PBMCs

Up to 3 cryovials, containing each 5 – 10 x 10^6^ PBMCs in freezing medium, were incubated at 37°C for 5 minutes and after thawing transferred to a 50 mL Falcon tube. Cell culture medium (RPMI-1640 supplemented with 10% FCS (GIBCO), 1% Penicillin/Streptomycin (GIBCO), and 1% GlutaMAX (GIBCO)) was added drop-wise up to a total volume of 50 mL, with a 1 min incubation and mixing step after each duplication of the volume. Following a spin (400 g, 5 min, 4°C) and removal of the supernatant, cell pellets were resuspended in 1 mL PBS and counted utilizing a Coulter counter, as described above.

### 4.4 Cell culture and drug screening

In the presented study, immunomodulatory drugs, which are in development to target epigenetic modifications, were used. After spinning, PBMCs were adjusted to a final concentration of 2 x 10^6^ cells/mL with cell culture medium, with or without supplementation of 750 ng/mL PHA-L (Roche) for cellular activation, and 100 µl cell suspension were seeded in 96-well plates. Compounds or DMSO were diluted in twofold of the desired concentrations in the cell culture medium, and 100 µl were directly added to the cells. PBMCs were cultured at 37°C, 5% CO^2,^ and 95% rH for 24 hours. Then 96-well plates were placed on ice for 10 minutes and cells were directly harvested in 1.4 mL FACS tubes. To prevent attachment of monocytes, the wells were additionally rinsed with PBS supplemented with 10 mM EDTA (Gibco).

### 4.5 Titration of viability dye and staining antibodies

To avoid antibody aggregates during measurements, antibody vials were centrifuged at 10.000 g for 5 min at RT prior first application. All reagents were titered using 250.000 thawed, unactivated PBMCs of the same donor per test in a final suspension volume of 100 µL. Prior to antibody staining, 1 µL Human TruStain FcX™ (BioLegend) per sample was added, carefully mixed, and cells were incubated for 10 min on ice. All antibodies were tested in a 2-fold serial dilution ranging from 20 µL to 0.25 µL per test, according to the manufacturer’s recommendations. For titration of the viability dye staining, PBMCs were spiked with dead cells. Therefore, PBMCs were heat-killed at 65°C for 10 minutes and mixed in a 1:1 ratio with viable cells. Live Dead Aqua dye was prepared according to the manufacturer’s recommendation, and titered in a 2-fold serial dilution ranging from 1:500 to 1:2000. PBMCs were stained for 30 min at 4°C in the dark, washed with PBS, and subsequently subjected to spectral flow cytometry measurements. Files were concatenated and analyzed in FlowJo (Version 10). For calculating the stain index/separation index (SI) (see Results section), for every antibody dilution values for the median fluorescence intensity of the positive and negative peak, respectively; as well as the standard deviation of the negative population were directly exported from FlowJo in .csv file format and SI was calculated and visualized in Excel.

### 4.6 Blocking and viability staining of PBMCs

For PBMC and T cell immunophenotyping, 300 000 PBMCs were stained in 1.4 mL FACS tubes in a final suspension volume of 100 µL. Reagents and antibodies were titrated beforehand, and staining protocols were optimized for ideal resolution and separation of all markers (details provided above, in the Results section and **Tables 3**, **4**). First, following the harvesting step and a spin (400 g, 5 min, 4°C), cell pellets were resuspended in 25 µL PBS. Then 1 µL Human TruStain FcX™ (BioLegend) per sample was added, carefully mixed, and cells were incubated for 10 min on ice. Next, Live Dead Aqua dye was prepared according to the manufacturer’s recommendations; diluted 1:250 in PBS, and 25 µL were added to each sample. Subsequently, samples were mixed and incubated for 10 min on ice. Afterwards, PBMCs were stained according to the PBMC and T cell immunophenotyping protocols, respectively.

**Table 3:** Reagents and dilutions used for the PBMC panel.

Fluorophore	Marker	Supplier	Clone	Catalogue number	Con. (µL/test)
BV421	CD126	BD Biosciences	M5	564163	1,25
eFlour450	HLA-DR	Thermo Fisher	L243	48-9952-42	1,25
BV480	IgD	Bd Bioscience	IA6-2	566138	0,5
L/D Aqua	Live/Dead	Thermo Fisher		L34957	0,1
BV570	CD16	BioLegend	3G8	302036	1,25
BV605	IgG	BD Biosciences	G18-145	563246	2,5
cFluorV610	CD4	Cytek	SK3	R7-20073	1,25
BV650	CD56	BioLegend	5.1H11	362532	0,5
BV711	CD95	BioLegend	DX2	305644	1,25
BV750	CD11b	BioLegend	M1/70	101267	1,25
BV785	PD-1	BioLegend	EH12.2H7	329930	1,25
BB515	CD11c	BD Biosciences	B-ly6	564490	1,25
FITC	CD69	BioLegend	FN50	310904	1,25
PE	CD27	BioLegend	O323	302808	1,25
PE-Dazzle594	CD70	BioLegend	113-16	355124	5
PerCp-Cy5.5	CD14	Thermo Fisher	61D3	45-0149-42	2,5
PE-Cy7	CD3	BioLegend	SK7	344816	0,5
APC	CD25	Thermo Fisher	BC96	17-0259-42	2,5
Alexa Flour647	CD169	BioLegend	7-239	346006	2,5
BD Horizon APC-R700	CD86	BD Bioscience	2331	565149	1,25
APC-Cy7	CD19	BioLegend	SJ25C1	363010	0,5
APC-Fire810	CD38	BioLegend	HIT2	303550	2,5

**Table 4:** Reagents and dilutions used for the T cell panel.

Fluorophore	Marker	Supplier	Clone	Catalogue number	Con. (µL/test)
BV421	CCR7	BioLegend	G043H7	353208	1,25
Pacific Blue	CD45RO	BioLegend	UCHL1	304216	2,5
BD Horizon 480	TCRγδ	BD Bioscience	11F2	746498	2,5
L/D Aqua	Live/Dead	Thermo Fisher		L34957	0,1
BV570	CD8a	BioLegend	RPA-T8	301038	0,5
BV605	CD134	BioLegend	Ber-ACT35	350028	1,25
BV650	CD28	BioLegend	CD28.2	302946	1,25
BV711	CCR6	BioLegend	G034E3	353436	1,25
BV750	CXCR5	BioLegend	J252D4	356942	1,25
BV785	PD-1	BioLegend	EH12.2H7	329930	1,25
FITC	CD69	BioLegend	FN50	310904	1,25
PE	CTLA-4	BioLegend	BNI3	369604	2,5
PE-Dazzle594	CXCR3	BioLegend	G025H7	353736	2,5
PerCP	CD45RA	BioLegend	HI100	304156	0,5
PerCP-Cy5.5	CD71	BioLegend	CY1G4	334114	2,5
PerCP-eFlour710	CCR4	Thermo Fisher	D8SEE	46-1949-42	1,25
PE-Cy7	CD4	BD Bioscience	SK3	557852	0,5
APC	CD25	Thermo Fisher	BC96	17-0259-42	2,5
Alexa Fluor 647	ICOS	BioLegend	C398.4A	313516	5
BD Horizon APC-R700	CD127	BD Bioscience	HIL-7R-M21	565185	1,25
APC-Cy7	CD3	BD Bioscience	SK7	557832	1,25
APC-Fire810	CD38	BioLegend	HIT2	303550	2,5

### 4.7 Staining for PBMC immunophenotyping

Following viability staining, as described above, an antibody cocktail was prepared for a total volume of 50 µL per sample containing all antibodies from the panel in the evaluated final concentrations (described in **Table 3**) diluted in PBS supplemented with 2% FCS. 50 µL of the antibody cocktail were added to each sample and incubated for 30 min at 4°C in the dark. Following washing with 1 mL PBS and a spin (400 g, 5 min, 4°C), pellets were resuspended in 70 µL PBS supplemented with 2% FCS and spectral flow cytometry measurements were performed subsequently.

### 4.8 Staining for T cell immunophenotyping

After viability staining, as described above, 1.25 µL of the BV750-CXCR5 antibody was added separately to each sample, carefully mixed, and incubated for 10 min at 4°C in the dark. Then, 1.25 µL BV785–PD1 per sample was applied, mixed, and incubated for another 10 min at 4°C in the dark. Subsequently, an antibody cocktail was prepared for a total volume of 47.5 µl per sample containing the remaining antibodies from the immunophenotyping panel in the evaluated final concentrations (described in **Table 4**) diluted in PBS supplemented with 2% FCS. 47.5 µL of the antibody cocktail were added to each sample and incubated for 30 min at 4°C in the dark. Following washing with 1 mL PBS and a spin (400 g, 5 min, 4°C), pellets were resuspended in 70 µL PBS supplemented with 2% FCS and spectral flow cytometry measurements were performed subsequently.

### 4.9 Spectral flow cytometer instrument setup

Spectral flow cytometry measurements were executed utilizing an Aurora spectral flow cytometer (Cytek), equipped with a 3-laser configuration (405 nm, 488 nm, and 640 nm). Prior to every measurement, calibration was performed using Cytek SpectroFlo^®^ QC beads (Cytek Biosciences). For spectral flow cytometry measurements the “gain settings” from the CytekAssaySetting of the SpectroFlo^®^ software (Cytek Biosciences) were used as a starting point, and the scatter profiles were optimized for PBMCs. All fluorescence signals were on scale and all tubes were recorded utilizing the same gain settings. The minimum forward scatter threshold was adjusted to 50 000 units to eliminate debris from the measurement. Events were recorded at a rate of fewer than 6 000 events per second and in total 250 000 events were acquired for every tube. After the measurement, the experimental file was “live unmixed” in the SpectroFlo^®^ software. For setting up the “unmixing” settings in the SpectroFlo^®^ wizard, single-stained PBMCs and single-stained UltraComp eBeads™ (Invitrogen, Thermo Fisher) were recorded (details for “unmixing” are provided in the Results section). Data were exported in .fcs file format.

### 4.10 Data import and clean up

Data were imported into FlowJo™ software (version 10) in. fcs file format. First, all samples were subjected to data cleanup using the FlowAI plugin in FlowJo. FlowAI was applied on all uncompensated parameters and FlowAI settings were as follows: Anomalies to exclude = Flow rate & dynamic range, Second fraction FR = 0.100, Alpha FR = 0.0100, Maximum changepoints = 3, Changepoint penalty = 200, Dynamic range check side = Both; in addition outliers were directly removed by the software. Using FlowAI, in all samples less than 5% of events were removed by the software. “Good events” calculated by the FlowAI software were used for further analysis.

### 4.11 Data analysis in FlowJo™

Following data cleanup, all samples were manually pre-gated to remove remaining aggregates, debris, and doublets by evaluating the scatter profiles (see Results section, **Figure 2**). Further manual gating for individual PBMC subsets and T cell populations, respectively, was performed in accordance with the literature and is displayed in **Figure 2**. Next, the proper scaling of the data were inspected in FowJo™ to ensure the positive and negative population of every fluorophore was stretched across the axis. In addition, dimensionality reduction was performed using the UMAP and tSNE plugins by FlowJo™. Before dimensionality reduction, paired samples were concatenated including metadata. First, to visualize main subpopulations in the samples of the PBMC immunophenotyping (**Figure 2B**), UMAP analysis was trained on viable cells utilizing the following compensated fluorescent parameters: HLA-DR, IgD, CD16, CD56, CD11b, CD11c, CD27, CD14, CD19, and CD3; and settings: Euclidean; Nearest Neighbors = 15; Minimum Distance = 0.5; Number of Components = 2. In samples of the T cell immunophenotyping, a UMAP of CD3^+^ cells was calculated utilizing CD4, CD8, TCRγδ, CCR7, and CD45RA; and settings as described above (**Figure 2E**). The UMAP analysis of CD4^+^ T cells was performed using CCR7, CCR6, CXCR5, CXCR3, CD45RA, CCR4, CD25 and CD127 (**Figure 2G**). For visualizing global alterations of the expression of activation markers by dimensionality reduction, tSNE analysis was trained on the viable population in the samples of the PBMC and T cell immunophenotyping, respectively. tSNE settings were as follows: all fluorescent parameters used besides Live/Dead, iterations = 1000, perplexity = 30, learning rate = 50400, KNN algorithm = Exact (vantage point tree), gradient algorithm = Barnes-Hut (**Figures 3C**, **4B**). From each specified subset, the percentages of the respective population and the median fluorescence intensity (MFI) of the individual activation marker expression in the depicted subsets were extracted and exported in .csv file format.

### 4.12 Data analysis and statistics in R

All subsequent analysis was performed in RStudio (R Development Core Team). Data from. csv files were imported into the R environment utilizing standard import functions. Heatmaps were created using the “Complex heatmap” R package, facet boxplots were generated utilizing the R package “ggplot”; for the 3-dimensional Principal Component Analysis (PCA) and the corresponding Loadings Plot the “plotly” R package was used (lower 10% of variables were removed based on variance); and for the bar charts “ggplot2” and “plotly” R packages were used. Statistical significance was calculated in RStudio using the “rstatix” R package. The fractional difference (FD) of every marker was calculated by dividing the marker median fluorescence intensity (MFI) of the compound by the respective marker MFI of the DMSO control and subtracting 1 (FD = MFI_compound_/MFI_DMSO_) – 1). The activation profile expression matrix for the 3-dimensional PCA was generated by integrating the min-max normalized values of every marker-cell type combination of the PBMC and T cell panel in one dataset. Except where otherwise indicated, statistical comparisons were done by one-sample t-test, comparing the 6 individual replicates of every condition to 0 (DMSO). Significance was defined as p-value (*P < 0.05, **P < 0.01, and ***P < 0.001).

## Data availability statement

The raw data supporting the conclusions of this article will be made available by the authors, without undue reservation.

## Ethics statement

Ethical approval for this study was granted by the ethics committee of the Medical University of Vienna, Austria (1073/2021; 1075/2021; 1448/2019). All research was conducted in compliance with fundamental ethical principles, as stated in the Charter of Fundamental Rights of the European Union (2010/C/83/2), in agreement with Horizon 2020 Ethics guidelines. The patients/participants provided their written informed consent to participate in this study.

## Author contributions

TP designed the research, performed most of the experiments, analyzed the data, and wrote the manuscript. LG coordinated the recruitment of PBMC donors performed some of the experiments and helped with the analysis of the datasets and wrote the manuscript. TP and MBr performed the bioinformatics analysis. MBo designed the research and wrote the manuscript. All authors contributed to the article and approved the submitted version.

## Supplementary material

Supplementary Figure 1 Panel design for the PBMC immunophenotyping panel **(A)** Readout of the Cytek Full Spectrum Viewer (Cytek Biosciences) displaying the spectral signatures of the 22 fluorophores in the 3L configuration of the Cytek Aurora (top). Optical layout of the used markers and fluorophores showing the approximate peak emission wavelengths (nm) (bottom). **(B)** Autofluorescence of unstained cells. Spectral fingerprint exported from the SpectroFlo^®^ software (Cytek Biosciences) of viable unstained non-activated PBMCs and unstained activated PBMCs of 1 representative healthy control.

Supplementary Figure 2 Similarity indices of the immunophenotyping panels. **(A, B)** Similarity Index Matrix of the **(A)** PBMC immunophenotyping panel and **(B)** T cell immunophenotyping panel obtained from the “Similarity™ & Complexity™” resources of the Cytek Full Spectrum Viewer (Cytek Biosciences). A value of “0” implies unique spectral signatures of the fluorophores; a value of “1” indicates fluorophores with identical signatures. The Complexity Index at the bottom of the matrix illustrates the overall complexity of all fluorophores in the respective panel.

Supplementary Figure 3 Comprehensive activation of different PBMC subsets by PHA. **(A)** Schematic illustration of the workflow. PBMCs of 6 healthy controls (HCs) were stimulated in the presence/absence of PHA for 24 hours. Immunophenotyping was performed subsequently. **(B, C)** Bar charts depicting the percentages of indicated subsets within **(B)** viable PBMCs (top), CD19^+^ B cells (bottom), **(C)** CD4^+^ T cells (left, top), CD8^+^ T cells (left, bottom), and CD4^+^ T cells (right, top). Each symbol indicates 1 independent biological sample. Statistical comparisons were done by Student’s t-test, comparing the PHA-stimulated (+) to the non-stimulated (-) condition of the individual PBMC subsets in 6 replicates. Significance was defined as p-value (*P < 0.05, **P < 0.01, and ***P < 0.001). **(D, E)** Summary heatmap depicting the marker expression as median fluorescent intensity (MFI) in the indicated **(D)** PBMC and **(E)** T cell subsets of 6 pooled HCs. Each column represents either the non-stimulated (-) or the PHA-stimulated (+) condition of every subset. Heatmaps were generated in R using the “Complex Heatmap” package; data were scaled by row and clustered by row. **(F, H)** Spectral flow cytometry analysis showing CD69 expression in the indicated **(F)** PBMC and **(H)** T cell subsets of 1 representative donor in the absence (-) and presence (+) of PHA, respectively. Numbers indicate the percentage of cells in the quadrants or gates. **(G, I)** Histograms showing **(G)** CD95 expression or **(I)** ICOS and PD-1 expression in the indicated **(G)** PBMC and **(I)** T cell subsets, respectively, in the non-stimulated (-, green) or PHA-stimulated (+, purple) condition. **(A–G)** The DC subset encompasses pDCs and mDCs. Data are representative **(F–I)** or show a summary **(B–E)** of at least 6 independent experiments. Abbreviations are used as follows: dendritic cells (DC), monocytes (Mos), natural killer cells (NKs), B cells (Bc), T cells (Tc), naïve (N), unswitched memory (UnMe), switched memory (SwMe), double-negative (DN), double-positive (DP), naïve T cells (TN), central memory T cells (TCM), terminally differentiated effector T cells (TEMRA), effector memory T cells (TEM), T helper cells (Th), regulatory T cells (Treg), T follicular helper cells (Tfh), T peripheral helper cells (Tph).

Supplementary Figure 4 Subset distribution. **(A)** High-dimensional data analysis using Uniform Manifold Approximation and Projection (UMAP) trained on CD4^+^ T cells of concatenated non-stimulated healthy controls depicting the accurate separation of the manually gated subsets. UMAPs were generated in FlowJo. **(B)** CTLA-4 expression on different T cell compartments. Spectral flow cytometry analysis depicting CTLA-4 expression on CD4^+^ CD25^+^, CD4^+^ CD25^-^ T cells and Th1 cells in 3 representative healthy controls after 24-hour cultivation without PHA. **(C, D)** Summary bar diagrams depicting the percentages of indicated **(C)** PBMC and (D) T cell subsets, respectively, activated for 24 hours in the presence of drug 1-5 or DMSO as control. Bar charts show percentages within **(C)** viable PBMCs or **(D)** CD3^+^ T cells (for CD4^+^ and CD8^+^ T cells) and CD4^+^ T cells (for Th1, Th2, Th17, Tfh, Tph and Treg), respectively. Statistical comparisons were done by one-sample t-test, comparing the 6 individual replicates of every condition to DMSO. Significance was defined as p-value (**P* < 0.05, ***P* < 0.01, and ****P* < 0.001). **(D)** The DC subset encompasses pDCs and mDCs. Abbreviations are used as follows: dendritic cells (DC), monocytes (Mo), natural killer cells (NKs), B cells (Bc), T cells (Tc), naïve T cells (TN), central memory T cells (TCM), terminally differentiated effector T cells (TEMRA), effector memory T cells (TEM), T helper cells (Th), regulatory T cells (Treg), T follicular helper cells (Tfh), T peripheral helper cells (Tph).

Supplementary Figure 5 Immunophenotyping of RA patients. **(A)** Summary heatmap depicting the marker expression as median fluorescent intensity (MFI) in the indicated (A) PBMC and (B) T cell subsets of 5 pooled RA patients and 5 pooled HCs, respectively. Each column represents the indicated subsets in either HCs or RA patients, each row depicts the marker expression. For the expression values of every marker - cell type combination a min-max normalization was applied. Heatmaps were generated in R using the “Complex Heatmap” package, k-means clustering was applied imposing **(A)** 4 clusters and **(B)** 5 clusters, respectively; data were scaled by row and clustered by rows. **(C)** Loadings plot for the corresponding PCA in depicting the individual component loadings of the marker – cell type combinations in PC1, PC2 and PC3. **(A–C)** The DC subset encompasses pDCs and mDCs. Abbreviations are used as follows: Principal Component Analysis (PCA), principal component (PC), healthy control (HC), rheumatoid arthritis (RA), dendritic cells (DC), monocytes (Mo), natural killer cells (NKs), B cells (Bc), T cells (Tc), T helper cells (Th), regulatory T cells (Treg), T follicular helper cells (Tfh), T peripheral helper cells (Tph).
