# Supplementary figures and images for: Advanced immunophenotyping: A powerful tool for immune profiling, drug screening, and a personalized treatment approach
Source: Front Immunol. 2023 Mar 24;14:1096096. doi: 10.3389/fimmu.2023.1096096 (PMC10080106; doi:10.3389/fimmu.2023.1096096)

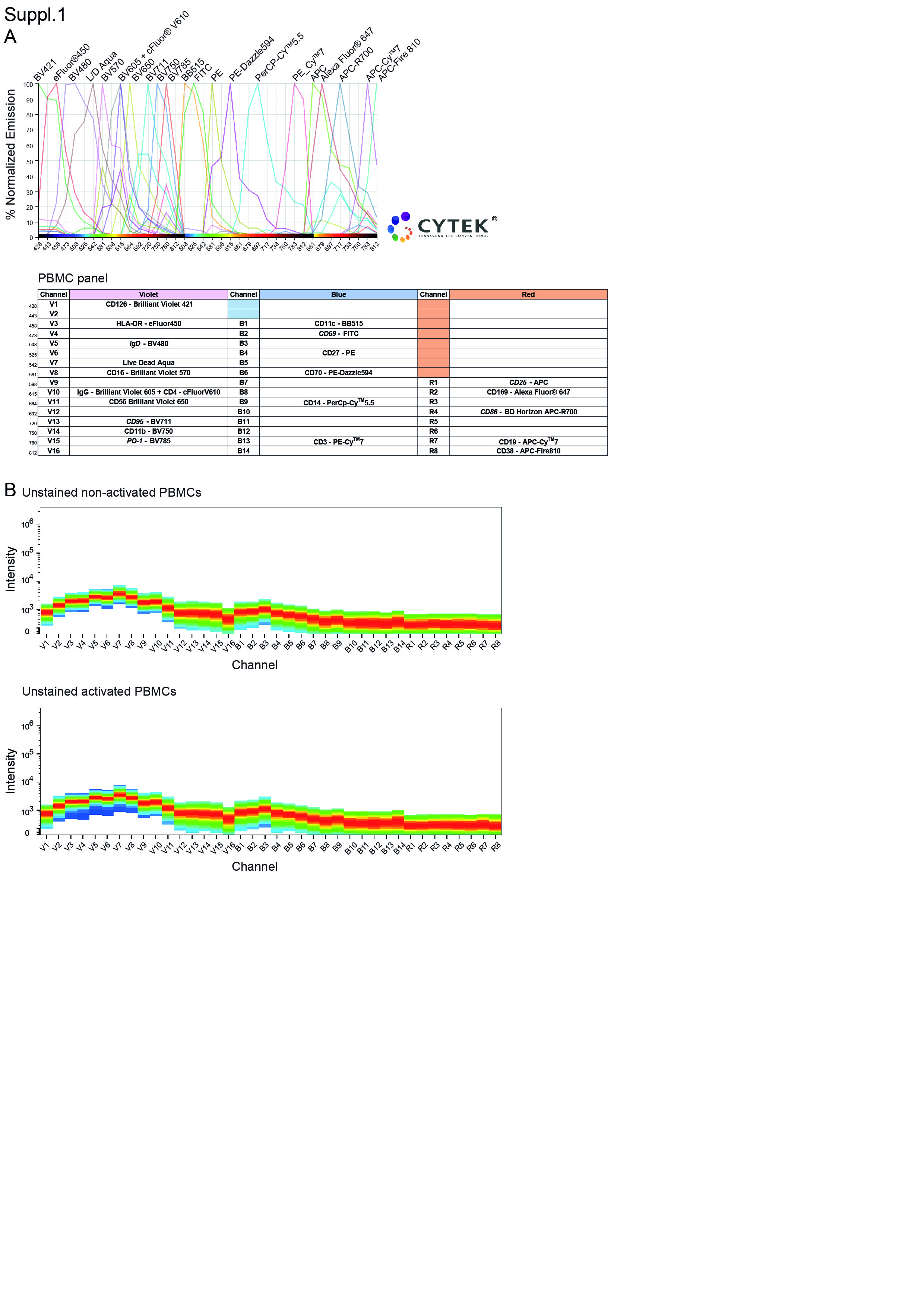

Supplement: Supplementary Figure 1 — Panel design for the PBMC immunophenotyping panel (A) Readout of the Cytek Full Spectrum Viewer (Cytek Biosciences) displaying the spectral signatures of the 22 fluorophores in the 3L configuration of the Cytek Aurora (top). Optical layout of the used markers and fluorophores showing the approximate peak emission wavelengths (nm) (bottom). (B) Autofluorescence of unstained cells. Spectral fingerprint exported from the SpectroFlo® software (Cytek Biosciences) of viable unstained non-activated PBMCs and unstained activated PBMCs of 1 representative healthy control. [file Image_1.jpg]

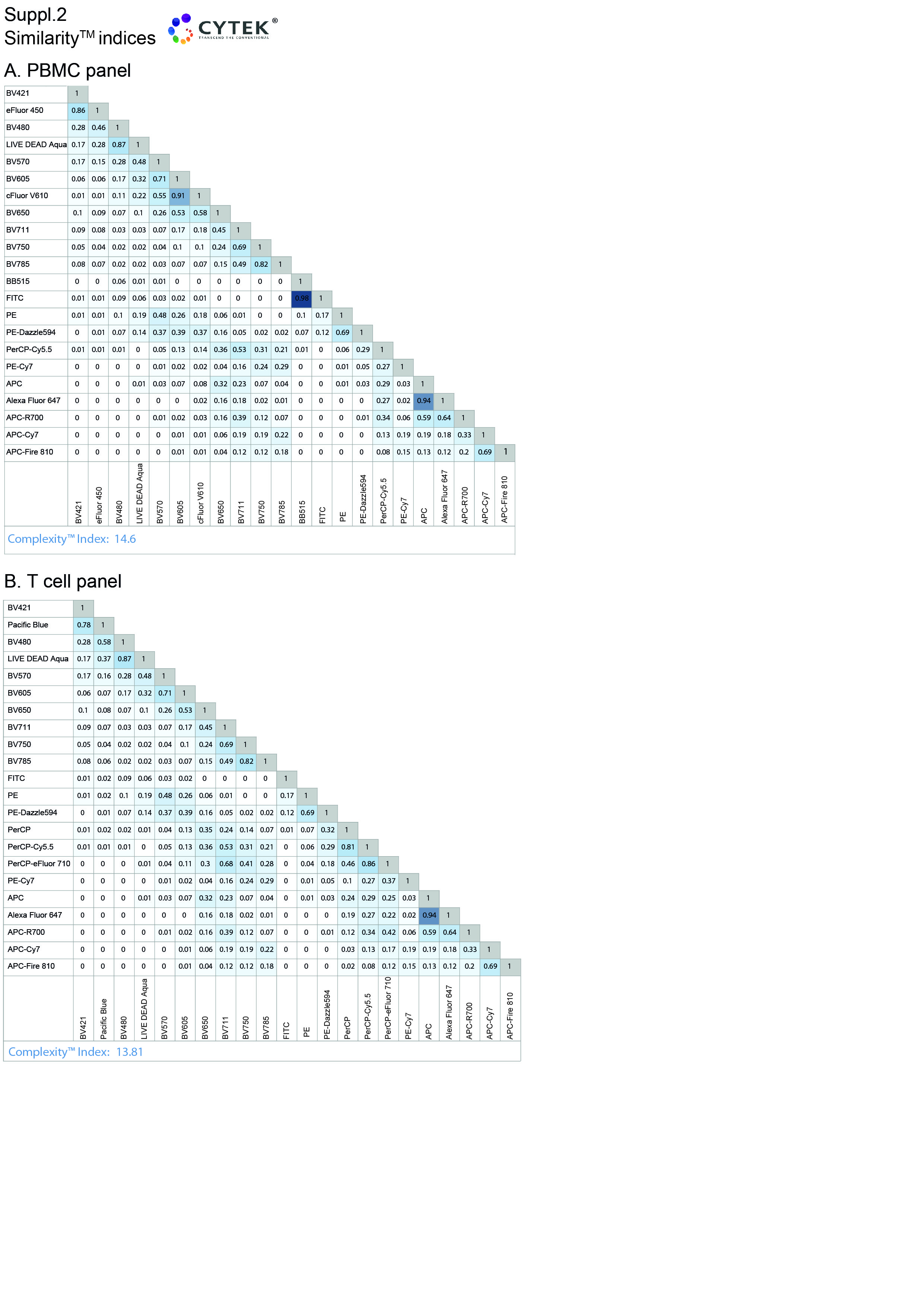

Supplement: Supplementary Figure 2 — Similarity indices of the immunophenotyping panels. (A, B) Similarity Index Matrix of the (A) PBMC immunophenotyping panel and (B) T cell immunophenotyping panel obtained from the “Similarity™ & Complexity™” resources of the Cytek Full Spectrum Viewer (Cytek Biosciences). A value of “0” implies unique spectral signatures of the fluorophores; a value of “1” indicates fluorophores with identical signatures. The Complexity Index at the bottom of the matrix illustrates the overall complexity of all fluorophores in the respective panel. [file Image_2.jpg]

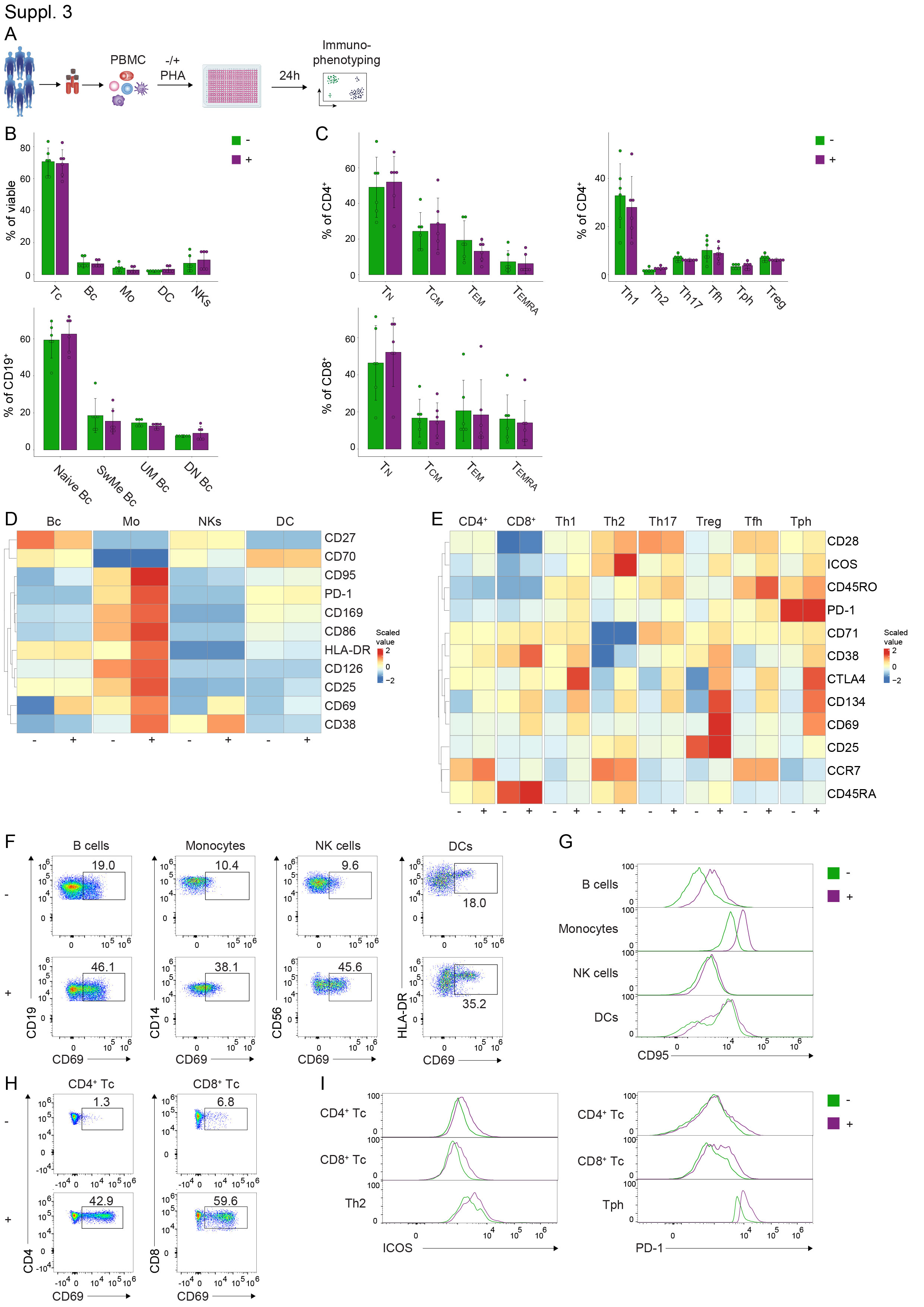

Supplement: Supplementary Figure 3 — Comprehensive activation of different PBMC subsets by PHA. (A) Schematic illustration of the workflow. PBMCs of 6 healthy controls (HCs) were stimulated in the presence/absence of PHA for 24 hours. Immunophenotyping was performed subsequently. (B, C) Bar charts depicting the percentages of indicated subsets within (B) viable PBMCs (top), CD19+ B cells (bottom), (C) CD4+ T cells (left, top), CD8+ T cells (left, bottom), and CD4+ T cells (right, top). Each symbol indicates 1 independent biological sample. Statistical comparisons were done by Student’s t-test, comparing the PHA-stimulated (+) to the non-stimulated (-) condition of the individual PBMC subsets in 6 replicates. Significance was defined as p-value (*P < 0.05, **P < 0.01, and ***P < 0.001). (D, E) Summary heatmap depicting the marker expression as median fluorescent intensity (MFI) in the indicated (D) PBMC and (E) T cell subsets of 6 pooled HCs. Each column represents either the non-stimulated (-) or the PHA-stimulated (+) condition of every subset. Heatmaps were generated in R using the “Complex Heatmap” package; data were scaled by row and clustered by row. (F, H) Spectral flow cytometry analysis showing CD69 expression in the indicated (F) PBMC and (H) T cell subsets of 1 representative donor in the absence (-) and presence (+) of PHA, respectively. Numbers indicate the percentage of cells in the quadrants or gates. (G, I) Histograms showing (G) CD95 expression or (I) ICOS and PD-1 expression in the indicated (G) PBMC and (I) T cell subsets, respectively, in the non-stimulated (-, green) or PHA-stimulated (+, purple) condition. (A–G) The DC subset encompasses pDCs and mDCs. Data are representative (F–I) or show a summary (B–E) of at least 6 independent experiments. Abbreviations are used as follows: dendritic cells (DC), monocytes (Mos), natural killer cells (NKs), B cells (Bc), T cells (Tc), naïve (N), unswitched memory (UnMe), switched memory (SwMe), double-negative (DN), double-positive (DP), naïve T [file Image_3.jpeg]

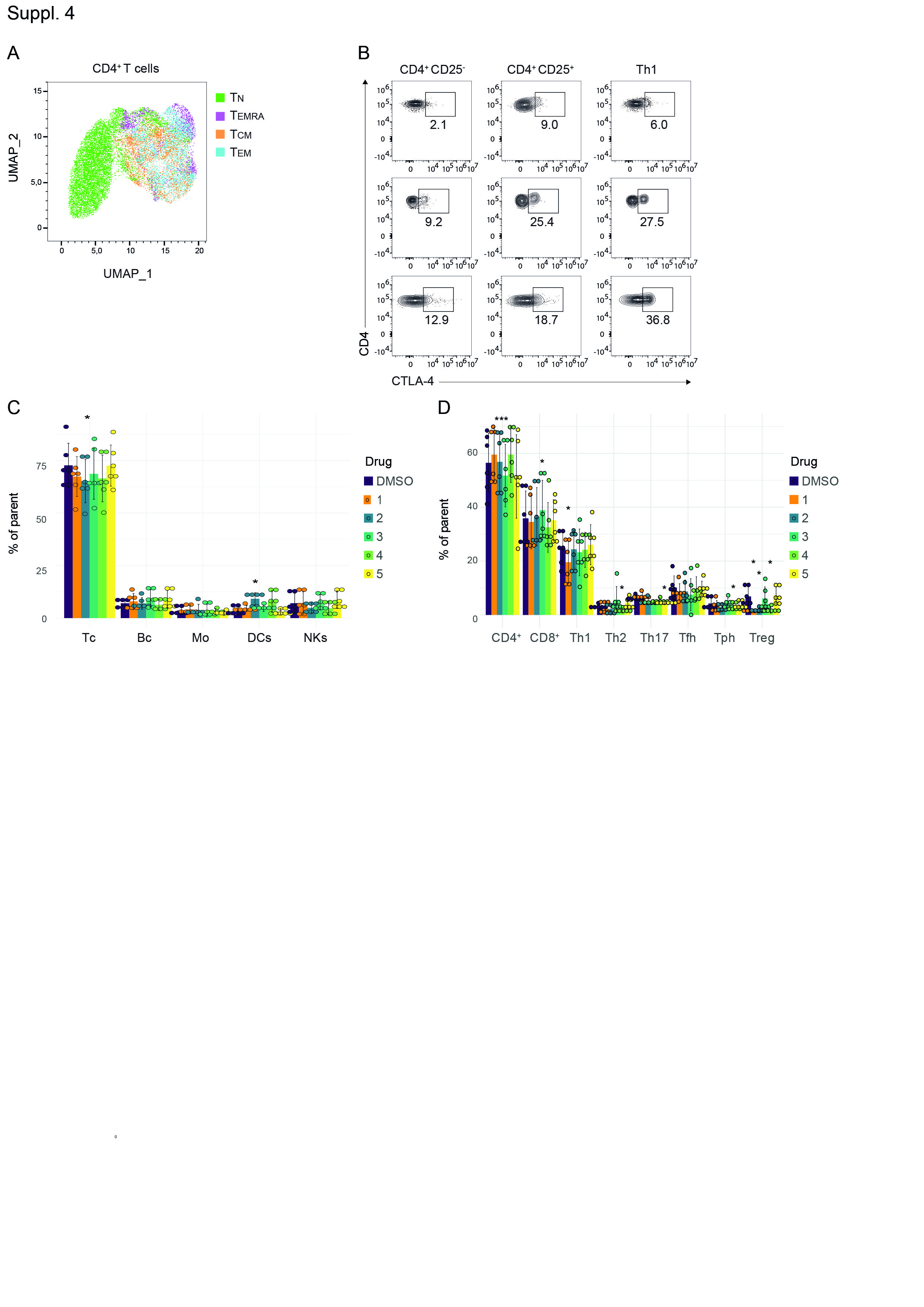

Supplement: Supplementary Figure 4 — Subset distribution. (A) High-dimensional data analysis using Uniform Manifold Approximation and Projection (UMAP) trained on CD4+ T cells of concatenated non-stimulated healthy controls depicting the accurate separation of the manually gated subsets. UMAPs were generated in FlowJo. (B) CTLA-4 expression on different T cell compartments. Spectral flow cytometry analysis depicting CTLA-4 expression on CD4+ CD25+, CD4+ CD25- T cells and Th1 cells in 3 representative healthy controls after 24-hour cultivation without PHA. (C, D) Summary bar diagrams depicting the percentages of indicated (C) PBMC and (D) T cell subsets, respectively, activated for 24 hours in the presence of drug 1-5 or DMSO as control. Bar charts show percentages within (C) viable PBMCs or (D) CD3+ T cells (for CD4+ and CD8+ T cells) and CD4+ T cells (for Th1, Th2, Th17, Tfh, Tph and Treg), respectively. Statistical comparisons were done by one-sample t-test, comparing the 6 individual replicates of every condition to DMSO. Significance was defined as p-value (*P < 0.05, **P < 0.01, and ***P < 0.001). (D) The DC subset encompasses pDCs and mDCs. Abbreviations are used as follows: dendritic cells (DC), monocytes (Mo), natural killer cells (NKs), B cells (Bc), T cells (Tc), naïve T cells (TN), central memory T cells (TCM), terminally differentiated effector T cells (TEMRA), effector memory T cells (TEM), T helper cells (Th), regulatory T cells (Treg), T follicular helper cells (Tfh), T peripheral helper cells (Tph). [file Image_4.jpeg]

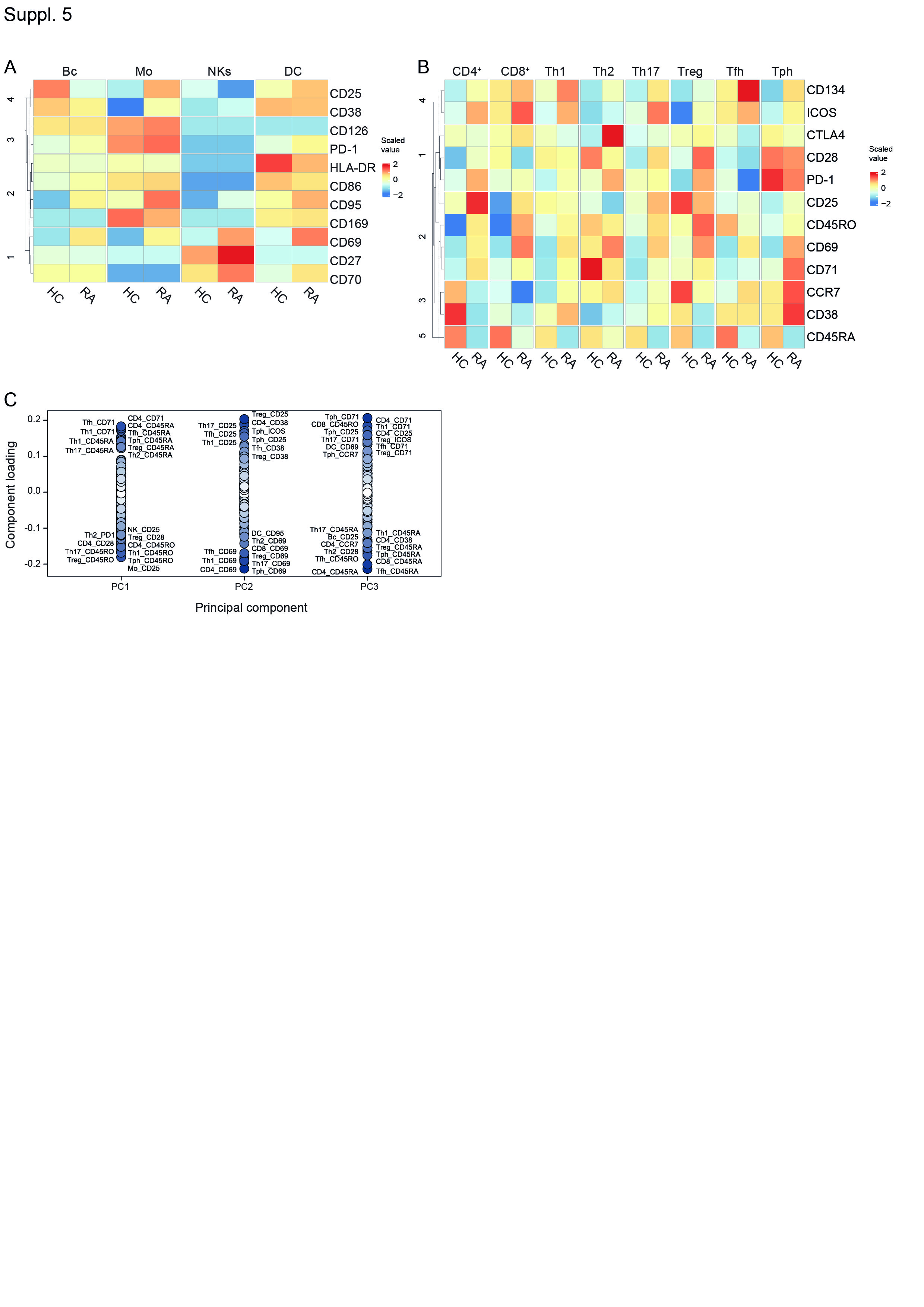

Supplement: Supplementary Figure 5 — Immunophenotyping of RA patients. (A) Summary heatmap depicting the marker expression as median fluorescent intensity (MFI) in the indicated (A) PBMC and (B) T cell subsets of 5 pooled RA patients and 5 pooled HCs, respectively. Each column represents the indicated subsets in either HCs or RA patients, each row depicts the marker expression. For the expression values of every marker - cell type combination a min-max normalization was applied. Heatmaps were generated in R using the “Complex Heatmap” package, k-means clustering was applied imposing (A) 4 clusters and (B) 5 clusters, respectively; data were scaled by row and clustered by rows. (C) Loadings plot for the corresponding PCA in depicting the individual component loadings of the marker – cell type combinations in PC1, PC2 and PC3. (A–C) The DC subset encompasses pDCs and mDCs. Abbreviations are used as follows: Principal Component Analysis (PCA), principal component (PC), healthy control (HC), rheumatoid arthritis (RA), dendritic cells (DC), monocytes (Mo), natural killer cells (NKs), B cells (Bc), T cells (Tc), T helper cells (Th), regulatory T cells (Treg), T follicular helper cells (Tfh), T peripheral helper cells (Tph). [file Image_5.jpeg]
